# The impact of sex and disease classification on patient-reported outcome measures in axial spondyloarthritis: a descriptive prospective cross-sectional study

**DOI:** 10.1186/s13075-019-2012-x

**Published:** 2019-10-29

**Authors:** Rikke A. Andreasen, Lars E. Kristensen, Kenneth Egstrup, Xenofon Baraliakos, Vibeke Strand, Hans Christian Horn, Inger M. J. Hansen, Robin Christensen, Torkell Ellingsen

**Affiliations:** 1 Department of Medicine, Section of Rheumatology, Odense University Hospital, Svendborg and University of Southern Denmark, Baagøes Allé 15, DK-5700 Svendborg, Denmark; 2 Musculoskeletal Statistics Unit, The Parker Institute, Copenhagen University Hospital, Bispebjerg and Frederiksberg Hospital, Copenhagen F, Denmark; 3 The DANBIO Registry, Centre for Rheumatology and Spine Diseases, Copenhagen, Denmark; 4 Cardiovascular Research Unit, Odense University Hospital, Svendborg, Denmark; 5 Rheumazentrum Ruhrgebiet Herne, Ruhr-University Bochum, Bochum, Germany; 6 Division of Immunology/Rheumatology, Stanford University, Palo Alto, CA USA; 7 Research Unit of Rheumatology, Department of Clinical Research, Odense University Hospital, University of Southern Denmark, Odense, Denmark; 8 OPEN, Odense Patient Data Explorative Network, Odense University Hospital, Odense, Denmark

**Keywords:** Spondyloarthritis, Ankylosing spondylitis, Quality of life, Patient-reported outcomes

## Abstract

**Background:**

The aim of this study was to explore the impact of sex and disease classification on outcomes in axial spondyloarthritis (axSpA) patients, including both radiographic (r-) axSpA and non-radiographic (nr-) axSpA, in males and females, respectively.

**Methods:**

AxSpA patients were consecutively recruited from two rheumatology outpatient university clinics. We explored how sex and axSpA disease classification affected patient-reported outcome measures (PROMs). General linear models were used to investigate if there was an association between the continuous variables and each of the main effects of interest (sex and axSpA classification), as well as the possible interaction between them. Categorical outcome measures were analyzed with the use of logistic regression with the same fixed effects. We analyzed the relationship between tender point count (TPC) and the Bath Ankylosing Spondylitis Disease Activity Index (BASDAI). The prevalence of extra-articular manifestations (EAMs) and the Charlson Comorbidity Index (CCI) were determined.

**Results:**

According to the protocol, a total of 100 outpatients with axSpA were enrolled (r-axSpA males 30, r-axSpA females 10, nr-axSpA males 25, nr-axSpA females 35). The BASDAI scores appeared higher among nr-axSpA females (median [Q_1_; Q_3_], 47 [21; 60]) compared with the combined median for the 3 other subgroups 25 [12; 25]. Female sex was associated with a higher number of tender point count (TPC, *P* < 0.001). TPC and BASDAI were correlated for female nr-axSpA patients (*r* = 0.44, *P* = 0.008) and male nr-axSpA patients (*r* = 0.56, *P* = 0.003). Being classified as nr-axSpA was associated with a lower SF-36 Mental Component Summary (median for the 4 subgroups: nr-axSpa females 46.7, nr-axSpA males 52.3 vs. r-axSpA males 56.9 and r-axSpA females 50.4). EAMs were frequent (up to 50%). The CCI was low in all 4 subgroups, and no difference in the CCI between the subgroups was observed (*P* = 0.14). However, male sex had a significant impact on the CCI (*P* = 0.03).

**Conclusions:**

In summary, patients with r-axSpA, regardless of sex, appeared less affected on most PROMs compared with nr-axSpA patients. However, female sex was associated with a higher number of TPC. TPC could possibly confound disease activity outcomes such as BASDAI, and one can consider different thresholds for defining high disease activity depending on the patient’s sex.

**Trial registration:**

The trial is registered and approved by the Region of Southern Denmark’s Ethics Committee (S-20150219). Registered 19 February 2015.

## Background

Spondyloarthritis (SpA) is a heterogeneous group of chronic rheumatic diseases; it can be dominated by peripheral joint involvement, classified as peripheral SpA (pSpA), or by inflammatory back pain, classified as axial SpA (axSpA). Furthermore, axSpA is currently subdivided into two groups: non-radiographic (nr-axSpA) and radiographic axSpA (r-axSpA) (also known as ankylosing spondylitis, AS). Next to the spinal and articular symptoms, many patients with axSpA also have extra-articular manifestations (EAMs, e.g., uveitis, enthesitis, psoriasis, inflammatory bowel disease) which contribute to reduced health-related quality of life [1]. Besides EAMs, axSpA patients have a higher risk of comorbidities [2–4].

AxSpA disorders are generally diagnosed more often in males than in females [5]. In reference to the most familiar form of these disorders, r-axSpA, three male cases are documented for every female case [5]. In contrast to r-axSpA, nr-axSpA patients show little difference in the prevalence among males and females [6].

Little is known of the differential clinical expression of axSpA between males and females [7, 8]. However, female axSpA patients are more likely to have shorter disease duration and less objective signs of inflammation but demonstrate a high burden of illness due to disease activity and impairment of health-related quality of life (HR-QoL) [9]. Despite some studies suggesting that this is due to the concomitant widespread pain syndrome (e.g., fibromyalgia), this notion as well as any speculation around cause and effect is still regarded as controversial [10, 11]. Few studies have analyzed sex differences in axSpA patients [7, 12, 13], and thus, there is a need to determine the actual distribution of these descriptive characteristics and the association with the self-reported burden of disease in a real-world setting (i.e., pragmatic and unbiased enrolment).

Our primary objective was to evaluate the impact of sex and axSpA disease classification on self-reported disease activity measures. Secondary objectives included evaluating whether there is a relationship between the continuous measure of muscular tender points [14] and the self-reported disease activity (e.g., Bath Ankylosing Spondylitis Disease Activity Index (BASDAI [15]), to determine the prevalence of EAMs and comorbidities, as defined by the CCI [16]. Finally, we compared the impact on HR-QoL measures in these axSpA patients in terms of how axSpA classification differentially affects aspects of physical, mental, and social well-being.

## Materials and methods

### Study design

As prespecified in the original protocol (Additional file 1) and elaborated in the statistical analysis plan (SAP, Additional file 2), the study was designed as a non-interventional descriptive prospective cross-sectional study, with consecutive enrolment of 100 representative axSpA patients. Information about the patients and their exposures were collected at a single center (Svendborg) at one visit according to the clinical standards in Denmark. All examinations were conducted on the same day. The inclusion period was between 1 April 2016 and 30 March 2018. Inadequate reporting of descriptive studies can be problematic for several reasons (e.g., as a consequence of post hoc reporting standards); however, existing reporting guidelines and checklists can help researchers achieve transparent reporting following the prespecified protocol [17]. Study findings are reported as a cross-sectional study according to *Strengthening The Reporting of OBservational Studies in Epidemiology* (STROBE) guidelines [18].

### Study participants

Patients referred with a likely axSpA diagnosis seen in the Department of Rheumatology, Odense University Hospital, in either Svendborg or Odense were informed about the project and recruited if eligible. Patients diagnosed with axSpA also fulfilled the ASAS classification criteria for axSpA (imaging arm) [19], and patients diagnosed with r-axSpA also fulfilled the modified New York criteria [20]. To be considered eligible, patients had had the ability and willingness to give written informed consent and to meet the requirements of the prespecified protocol. The only two exclusion criteria were if the patient did not understand Danish or not yet adults (age < 18 years). Briefly, patients should have had inflammatory back pain for ≥ 3 months and age at onset of < 45 years and were required to have sacroiliitis on imaging (MRI or X-ray) and ≥ 1 SpA feature (i.e., arthritis, enthesitis, dactylitis, uveitis, psoriasis, inflammatory bowel disease, and HLA-B27 [21]).

### Data collection

After the participants signed the informed consent, the following information was collected at the study visit: information on demographic characteristics, disease characteristics, medication history, types of axSpA features, patient-reported outcome measures (PROMs), and finally an assessment of comorbidities combined into the Charlson Comorbidity Index (CCI) [16].

All the core outcome domains were included as recommended by the ASAS/OMERACT initiatives [22], measured using the following outcome measurement instruments: physical function (the Bath Ankylosing Spondylitis Functional Index (BASFI) [15]), based on visual analog scales (VAS, range 0–100 mm), pain (VAS, range 0–100 mm), spinal mobility (BASMI, range 0–100 [23]), patient’s global assessment (PGA) [21] (VAS, range 0–100 mm), peripheral joints/entheses (44-joint count/the Spondyloarthritis Research Consortium of Canada [SPARCC] Enthesitis Index, range 0–16 [21, 24]), acute phase reactants (C-reactive protein (CRP) [mg/L]), and fatigue (VAS, range 0–100 mm).

Touchscreen questionnaires were used for PROMs at the inclusion visit [25]. HR-QoL was addressed using the Medical Outcomes Study 36-Item Short-Form Health Survey (SF-36): the Danish v. 2 of SF-36 which uses a 4-week recall period [26]. The eight domains are further summarized into physical component summary (PCS) and mental component summary (MCS) scores. PCS and MCS are norm-based scores with a mean of 50 and a standard deviation of 10, higher scores indicating better HR-QoL [27].

### Visualization by spydergrams

To compare HR-QoL states between the four phenotypes of axSpA patients, we used spydergram representations based on the SF-36 questionnaire [28]. The eight domains of SF-36 have been validated in rheumatic diseases [29–31]; these include limitations in physical activities because of health problems (physical function), limitations in usual role activities because of physical health problems (role physical), bodily pain, general health perceptions, vitality, limitations in social activities because of physical or emotional problems (social functioning), limitations in usual role activities because of emotional problems (role emotional), and psychological distress and well-being (mental health index).

We used “spydergrams” which provide a visual method to examine the eight domains of HR-QoL scored from 0 (worst) to 100 (best) simultaneously in a single figure [28]. Danish normative data were used for comparison (32). Using spydergrams enabled us to compare the impact on HR-QoL in axSpA patients, specifically in terms of how axSpA classification differentially affects aspects of physical, mental, and social well-being.

We used the Ankylosing Spondylitis Disease Activity Score (ASDAS) including C-reactive protein (CRP) as a measure of axSpA disease activity. ASDAS-CRP has validated cutoffs and is endorsed by the Assessment of SpondyloArthritis international Society (ASAS) and Outcome Measures in Rheumatology (OMERACT) [21].

Blood samples were collected by a trained laboratory technician and treated according to set procedures. Results on inflammation (C-reactive protein [CRP], plasma calprotectin), human leukocyte antigen B27 (HLA-B27), and fecal calprotectin were collected. Calprotectin was analyzed by an ELISA kit from Calprotectin EliA™ (Thermo Fischer Scientific, Denmark [32]).

### Involvement of patient research partners and ethics approval

Collaboration between patient research partners (PRPs) and health care professionals in developing and disseminating research projects is relatively new. This project followed the EULAR recommendations [33]. The study was designed with the assistance from two Danish PRPs (Bent Duerlund [BD] and Lars Kruse Fischer [LKF]). The study was conducted according to the guidelines for good clinical practice, and the study protocol was approved by the Region of Southern Denmark’s Ethics Committee, approval number S-20150219).

### Statistical methods

As described in the original protocol (Additional file 1) and the SAP (Additional file 2), the anticipated analyses were outlined before reviewing the actual data. The characteristics of the participants were described for each sex (male or female) and axSpA classification (r-axSpA or nr-axSpA) using descriptive statistics: medians and interquartile ranges (IQRs) for continuous data and absolute numbers with corresponding percentages for binary outcomes were presented.

Despite this study being designed as a descriptive cross-sectional study, a few tests were applied. Differences between the groups were analyzed using Fisher’s exact test for categorical data and the Kruskal-Wallis *H* test for continuous data. Further, general linear models (GLM) were used to investigate if there was an association between the continuous variables (e.g., BASDAI) and each of the main effects of interest (sex and axSpA classification, respectively), as well as the possible interaction between them (Sex*Classification). Thus, the model included the following class variables (i.e., covariates): sex, axSpA disease classification, and the interaction between them as fixed effects. Categorical outcome measures were analyzed with the use of logistic regression with the same fixed effect covariates.

The relationship between the continuous measure of tender point count and the patient-reported disease activity score (BASDAI) was explored by scatter plots and analyzed with Spearman rank correlation coefficients; for consistency in the presentation, these were stratified on sex and axSpA classification as well. Prediction ellipses were added to give a visual impression of the distribution of data with the center representing the sample mean, i.e., a skinny ellipse indicates highly correlated variables, whereas an ellipse that is nearly circular indicates little correlation [34]. Data were analyzed using STATA version 15: level of statistical significance and use of confidence intervals. All applicable statistical tests were two-sided and were being performed using a 5% significance level. As multiplicity issues following the use of many statistical comparisons in a study like this increase the likelihood that a chance association could appear as potentially important, we only considered *P* < 0.05 to be indicative of a possible association as a consequence of the broad exploratory setting.

## Results

### Demographics

**Fig. 1:**
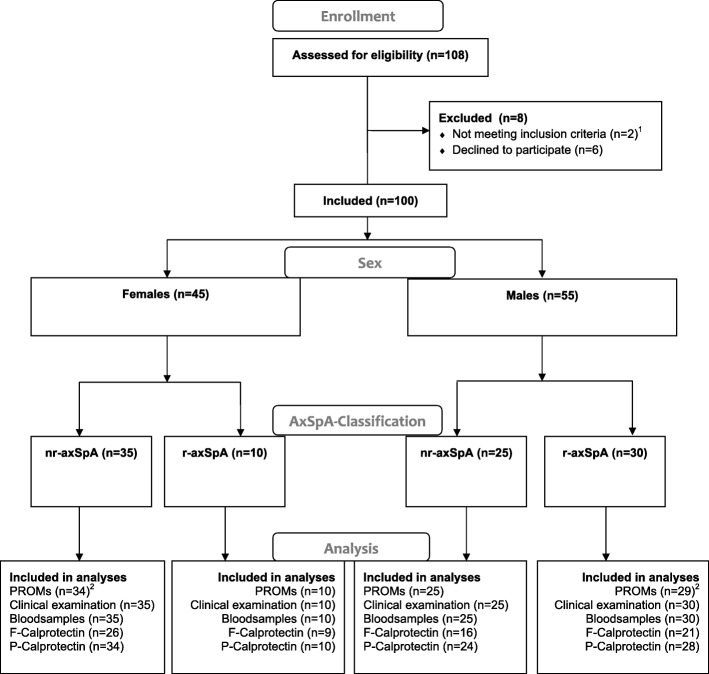
Numbers of axSpA patients, who were screened, included in the study and included in the analyses. Flow chart template modified from http://consort-statement.org. PROMS, patient-reported outcome measurements; F-calprotectin, fecal calprotectin; P-Calprotectin, plasma calprotectin. ^1^Did not fulfill the axSpA classification criteria, ^2^The Medical Outcomes Study SF-36 questionnaire is missing

As illustrated in Fig. 1, in order to get to a total of 100 AxSpA patients, we screened 108 axSpA patients referred to the clinic. Six of the referred individuals (5.6%), despite fulfilling the eligibility criteria, decided not to participate. Two of the 108 referred individuals (1.9%) did not fulfill the axSpA classification criteria when being examined by a trained rheumatologist.

For the total sample of 100 eligible patients, their overall demographic and clinical characteristics are summarized in Table 1. Forty patients were classified as r-axSpA and 60 as nr-axSpA (*P* = 0.05). There were more males (30.0%) classified as r-axSpA compared with females (10.0%, *P* < 0.001) corresponding to a male-to-female ratio of 3:1. Being classified as r-axSpA was associated with a significantly longer symptom duration (r-axSpA males 233 months and r-axSpA females 192 months vs. nr-axSpA females 90 months and nr-axSpA males 78 months, *P* < 0.001). However, for symptom duration, no significant interaction was observed between the type of axSpA diagnosis and sex (*P* = 0.79), and there was no difference between the sexes either (*P* = 0.84). r-axSpA patients were older than nr-axSpA patients (*P* = 0.04), and up to 44% of patients were current smokers among the entire axSpA group, with some variation across the 4 subgroups (*P* = 0.06). The body mass index (BMI) was higher in male axSpA patients compared with females (*P* = 0.002). BMI was not associated with axSpA classification (*P* = 0.12) and no interaction between sex and axSpA classification either (*P* = 0.21).

**Table 1:** Clinical characteristics and patient-reported outcome measures

	r-axSpA *n* = 40		Nr-axSpA *n* = 60		*P*	*P* (Sex)	*P* (AxSpA)	*P* (AxSpA*Sex)
	Male (*n* = 30)	Female (*n* = 10)	Male (*n* = 25)	Female (*n* = 35)				
Demographics								
Age, years, median [Q_1_; Q_3_]	48 [41; 55]	46 [38; 62]	40 [29; 51]	48 [36; 52]	0.18^a^	0.39	0.04	0.67
BMI, kg/m^2^, median [Q_1_; Q_3_]	25.7 [23.2; 30.9]	22.7 [20.6; 24.8]	28.1 [23.3; 30.2]	24.5 [23.3; 28.8]	0.02^a^	0.002	0.12	0.21
Smoking (current), *n* (%)	8 (27)	0 (0)	11 (44)	12 (34)	0.06^b^	0.73	0.29	0.85
Symptom duration, month, median [Q_1_; Q_3_]	233 [132; 384]	192 [132; 336]	78 [60; 120]	90 [48; 152]	< 0.001^a^	0.84	< 0.001	0.79
Peripheral joint involvement, *n* (%)	17 (57)	7 (70)	12 (48)	17 (49)	0.62^b^	0.73	0.29	0.85
Medication								
NSAIDs daily use, *n* (%)	12 (40)	5 (50)	13 (52)	17 (49)	0.82^b^	0.79	0.94	0.55
MTX use, *n* (%)	2 (7)	4 (40)	3 (12)	11 (31)	0.02^b^	0.09	0.61	0.40
MTX dose (mg/week),median [Q_1_; Q_3_]	0.0 [0.0; 10.0]	0.0 [0.0; 20.0]	0.0 [0.0; 0.0]	0.0 [0.0; 10.0]	0.005^a^	< 0.001	0.32	0.31
Sulfasalazine use, *n* (%)	1 (3)	1 (10)	1 (4)	4 (11)	0.48^b^	0.33	0.90	0.98
No. of previous bDMARD used, median [Q_1_; Q_3_]	0.0 [0.0; 1.0]	0.0 [0.0; 1.0]	0.0 [0.0; 1.0]	0.0 [0.0; 1.0]	0.94^a^	0.89	0.93	0.38
Current bDMARD, *n* (%)	15 (50)	3 (30)	12 (48)	10 (29)	0.25^b^	0.13	0.93	0.99
Glucocorticoid use, *n* (%)	0 (0)	1 (10)	0 (0)	0 (0)	0.10^b^	0.02	0.02	0.21
Glucocorticoid use (mg/day), median [Q_1_; Q_3_]	0.0 [0.0; 0.0]	0.0 [0.0; 0.0]	0.0 [0.0; 0.0]	0.0 [0.0; 0.0]	0.03^a^	0.02	0.02	0.02
Patient-reported outcome measures (PROMs)								
BASDAI (0–100), median [Q_1_; Q_3_]	25 [13; 45]	25 [16; 35]	25 [10; 52]	47 [21; 60]	0.07^a^	0.30	0.11	0.29
BASFI (0–100), median [Q_1_; Q_3_]	18 [7; 40]	19.0 [3; 49]	16 [7; 48]	36 [16; 51]	0.37^a^	0.47	0.34	0.57
SF-36: PCS (0–100), median [Q_1_; Q_3_]^†^	43.3 [37.0; 52.0]	51.3 [45.4; 54.5]	43.8 [37.1; 50.4]	43.4 [34.4; 46.7]	0.22^a^	0.83	0.29	0.10
SF-36: MCS (0–100), median [Q_1_; Q_3_]^†^	56.9 [50.1; 59.3]	50.4 [38.7; 60.4]	52.3 [39.1; 55.4]	46.7 [42.4; 52.2]	0.005^a^	0.09	0.03	0.21
VAS pain (0–100 mm), median [Q_1_; Q_3_]	24 [12; 43]	17 [12; 36]	25 [13; 47]	49 [20; 61]	0.10^a^	0.79	0.06	0.57
VAS fatigue (0–100 mm), median [Q_1_; Q_3_]	22 [12; 45]	39 [16; 54]	33 [12; 67]	54 [20; 61]	0.02^a^	0.08	0.16	0.38
VAS global (0–100 mm), median [Q_1_; Q_3_]	26 [14; 45]	19 [12; 57]	20 [12; 57]	53 [22; 69]	0.25^a^	0.39	0.30	0.41
Clinical examination								
Tender point count (0–18), median [Q_1_; Q_3_]	0 [0; 2]	4 [0; 6]	0 [0; 4]	5 [2; 8]	< 0.001^a^	< 0.001	0.28	0.24
Swollen joint count (0–44), median [Q_1_; Q_3_]	0 [0; 0]	0 [0; 0]	0 [0; 0]	0 [0; 0]	0.67^a^	0.89	0.32	0.23
Tender joint count (0–44), median [Q_1_; Q_3_]	0 [0; 2]	2 [0; 4]	0 [0; 2]	2 [1; 4]	0.02^a^	0.07	0.67	0.74
BASMI (0–100), median [Q_1_; Q_3_]	20 [10; 40]	10 [0; 30]	10 [10; 20]	10 [0; 20]	0.006^a^	0.12	0.02	0.30
SPARCC, EI (0–16), median [Q_1_; Q_3_]	0 [0; 2]	0 [0; 2]	1 [0; 2]	1 [0; 1]	0.05^a^	0.33	0.09	0.33
ASDAS-CRP, median [Q_1_; Q_3_]	2.0 [1.3; 3.1]	1.95 [1.3; 3.2]	2.1 [1.5; 3.1]	2.6 [1.4; 3.1]	0.95^a^	0.76	0.88	0.98
VAS physician (0–100), median [Q_1_; Q_3_]	11 [5; 21]	11 [9; 16]	10 [2.0; 17.0]	5 [2; 13]	0.63^a^	0.24	0.42	0.57
Extra-articular manifestations*								
Uveitis, *n* (%)	12 (40)	3 (30)	3 (12)	7 (20)	0.09^b^	0.42	0.51	0.33
Inflammatory bowel disease, *n* (%)	3 (10)	0 (0)	3 (12)	6 (17)	0.63^b^	0.58	0.81	0.58
Psoriasis, *n* (%)	12 (40)	2 (20)	11 (44)	14 (40)	0.64^b^	0.76	0.26	0.42
Dactylitis, *n* (%)	6 (20)	3 (30)	6 (24)	11 (31)	0.75^b^	0.53	0.93	0.87
Acilles enthesitis, *n* (%)	13 (43)	5 (50)	3 (12)	10 (29)	0.03^b^	0.14	0.21	0.43
Nephrolithiasis, *n* (%)	6 (20)	0 (0)	3 (12)	4 (11)	0.50^b^	0.95	0.43	0.49
Comorbidities								
Charlson Comorbidity Index, median [Q_1_; Q_3_]	1 [0; 1]	0 [0; 0]	1 [0; 1]	1 [0; 1]	0.14^a^	0.03	0.83	0.52
Paraclinical assessment								
HLA-B27 positive, *n* (%)	27 (90)	9 (90)	15 (60)	19 (54)	0.004^b^	0.66	0.06	0.86
CRP (mg/L), median [Q_1_; Q_3_]	4.7 [1.1; 10.0]	4.25 [1.1; 21]	2.1 [0.9; 6.8]	1.9 [0.6; 4.4]	0.13^a^	0.49	0.02	0.58
P-calprotectin (ng/mL), median [Q_1_; Q_3_]^††^	219 [145; 350]	144 [120; 405]	237 [137; 375]	195 [104; 288]	0.48^a^	0.14	0.66	0.77
F-calprotectin (mg/kg), median [Q_1_; Q_3_]^†††^	15 [10; 42]	16 [14; 27]	14 [7; 34]	19 [11; 55]	0.64^a^	0.79	0.91	0.24

Data are presented as median, 25th percentile (Q_1_) and 75th percentile (Q_3_), and percentages

*BMI* body mass index, *NSAIDs* nonsteroidal anti-inflammatory drugs, *MTX* methotrexate, *bDMARD* biologic disease-modifying drugs, *BASDAI* Bath Ankylosing Spondylitis Disease Activity Index, *BASFI* Bath Ankylosing Spondylitis Functional Index, *VAS* Visual Analog Scale, *SF-36*: *MCS/PCS* Medical Outcomes Study Short Form 36 Mental/Physical Component Summary, *BASMI* Bath Ankylosing Spondylitis Metrology Index, *SPARCC* Spondyloarthritis Research Consortium of Canada, *EI* Enthesitis Index, *ASDAS-CRP* Ankylosing Spondylitis Disease Activity Score-C-reactive protein

^a^Kruskal-Wallis *H* test

^b^Fisher’s exact test

*Either patient history or current diagnosis, P/F-calprotectin, plasma/fecal calprotectin

^†^*n* = 98 (r-axSpA male *n* = 29, r-axSpA female *n* = 10, nr-axSpA male *n* = 25, nr-axSpA female *n* = 34)

^††^*n* = 96 (r-axSpA male *n* = 28, r-axSpA female *n* = 10, nr-axSpA male *n* = 24, nr-axSpA female *n* = 34)

^†††^*n* = 72 (r-axSpA male *n* = 21, r-axSpA female *n* = 9, nr-axSpA male *n* = 16, nr-axSpA female *n* = 26)

The proportion of patients using methotrexate (MTX) differed among the subgroups (*P* = 0.02), but we found no evidence to suggest that sex (*P* = 0.09) or axSpA classification (*P* = 0.61) had an impact. However, the doses of methotrexate (MTX) were higher among the female axSpA patients compared with males (*P* < 0.001). Only one patient (a woman with r-axSpA) used current daily glucocorticoids.

### Patient-reported outcome measures, clinical examination, and paraclinical assessment

Exploring the impact of sex and axSpA disease classification on BASDAI, the scores appeared to be higher among the nr-axSpA females compared with nr-axSpA males, r-axSpA males, and r-axSpA females. Median [Q_1_; Q_3_] BASDAI was 47 [21; 60] for nr-axSpA females, and the combined median for the three other subgroups was 25 [12; 25]. However, we found no evidence to suggest that BASDAI was associated with sex (*P* = 0.30) or axSpA classification (*P* = 0.11). Being classified as nr-axSpA was associated (*P* = 0.03) with significantly lower SF-36 MCS (SF-36 MCS for the four subgroups: nr-axSpa females 46.7 and nr-axSpA males 52.3 vs. r-axSpA males 56.9 and r-axSpA females 50.4, *P* = 0.005). However, for MCS, no significant interaction was observed between sex and axSpA classification (*P* = 0.21) and no differences between the sexes (*P* = 0.09).

The level of fatigue differed across the subgroups (*P* = 0.02) but was not associated with sex (*P* = 0.08) and the type of axSpA diagnosis (*P* = 0.16) and no interaction either (*P* = 0.38). Exploring the impact of sex and axSpA disease classification on pain scores, the VAS scores could potentially be perceived as higher among the nr-axSpA females, compared with the other subgroups, but we found no association with sex (*P* = 0.79) or axSpA classification (*P* = 0.06). For the four subgroups, VAS_pain_: nr-axSpA females 49 and nr-axSpA males 25 vs. r-axSpA males 24 and r-axSpA females 17 respectively (*P* = 0.10). The number of swollen joints was equally low in all subgroups and was not associated with sex (*P* = 0.89) or axSpA classification (*P* = 0.32). The number of tender joint counts (TJC) differed across the subgroups (*P* = 0.02), and it appeared as if female patients had higher TJC, but we found no evidence to suggest TJC was associated with sex (*P* = 0.07) and axSpA classification (*P* = 0.67) and no interaction either (*P* = 0.74).

The tender point count (TPC) differed significantly between the subgroups (*P* < 0.001), and TPC was associated with female sex (*P* < 0.001), but not with axSpA classification (*P* = 0.28). We observed no interaction between sex and axSpA classification (*P* = 0.24). Patients classified as nr-axSpA had less-impaired spinal mobility compared with r-axSpA patients (*P* = 0.02), but no interaction was observed between the type of axSpA diagnosis and sex (*P* = 0.30), and there was no difference between the sexes either (*P* = 0.12).

Disease activity as determined by ASDAS-CRP did not differ significantly across the subgroups (*P* = 0.95) and was not associated with sex (*P* = 0.76) or axSpA classification (*P* = 0.88). However, nr-axSpA females had a median ASDAS-CRP of 2.6 corresponding to a high disease activity, despite a low CRP, whereas the other subgroups had low disease activity (ASDAS-CRP < 2.1). The median levels of CRP were higher in r-axSpA patients compared with nr-axSpA patients (*P* = 0.02), but no interaction was observed between axSpA classification and sex (*P* = 0.58) and no differences between the sexes either (*P* = 0.49).

The proportion of HLA-B27 positive was significantly different across the subgroups (*P* = 0.004). However, the evidence to suggest HLA-B27 to be associated with axSpA classification was vague (*P* = 0.06), no association with sex (*P* = 0.66), and no interaction either (*P* = 0.86).

The level of fecal calprotectin levels was not elevated (> 50 mg/kg) in our axSpA patients, and no differences in fecal calprotectin across the subgroups were observed (*P* = 0.64): sex (*P* = 0.79) and axSpA classification (*P* = 0.91) did not have an impact. The plasma calprotectin levels were also comparable across the four different groups—not associated with sex (*P* = 0.14) or axSpA classification (*P* = 0.66).

### Association between tender points and BASDAI

**Fig. 2:**
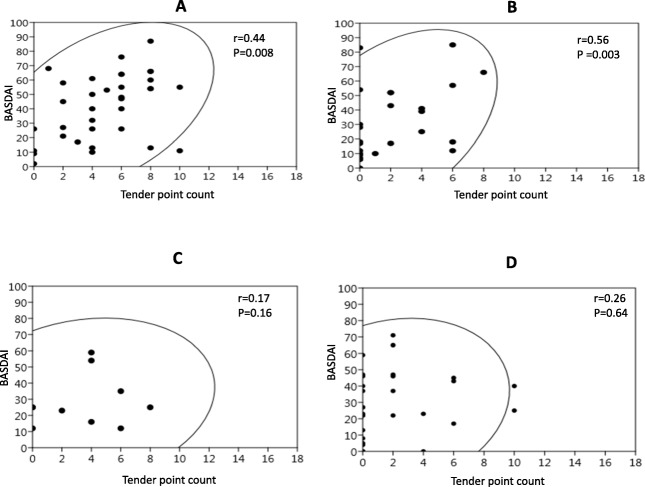
Association between tender point counts and BASDAI scores stratified on sex and axSpA classification. **a** Nr-axSpA females. **b** Nr-axSpA males. **c** r-axSpA females. **d** r-axSpA males

As illustrated in Fig. 2, we found a moderate positive correlation between TPC (ranging 0–18) and BASDAI for the 60 nr-axSpA patients (*r* = 0.44, *P* = 0.008, for females and *r* = 0.56, *P* = 0.003, for males). Even if there was a weak positive association between the two variables for the patients classified as r-axSpA (including only 40 patients), the smaller correlation coefficient did not reach statistical significance (*r* = 0.17, *P* = 0.16, for females and *r* = 0.26, *P* = 0.64, for males).

### Extra-articular manifestations and comorbidity

EAMs were frequent and, with the exception of a history of Achilles enthesitis, equally prevalent in r-axSpA and nr-axSpA patients. The most common EAMs were psoriasis (prevalence 20–44%), Achilles enthesitis (prevalence 12–50%), and uveitis (prevalence 12–40%). The proportion with inflammatory bowel disease (IBD) among nr-axSpA females (17%) was more similar to the nr-axSpA male (12%) and r-axSpA male (10%) groups than the r-axSpA female group (0%). However, sex and axSpA classification did not have an impact on EAM prevalence.

The burden of comorbidity assessed by the Charlson Comorbidity Index (CCI) was generally low in our axSpA population (median CCI for the four subgroups: r-axSpA males, 1; r-axSpA females, 0; nr-axSpA males, 1; nr-axSpA females, 0), and no differences between the subgroups were found (*P* = 0.14). However, male sex had a significant impact on the CCI (*P* = 0.03).

### Health-related quality of life

**Fig. 3:**
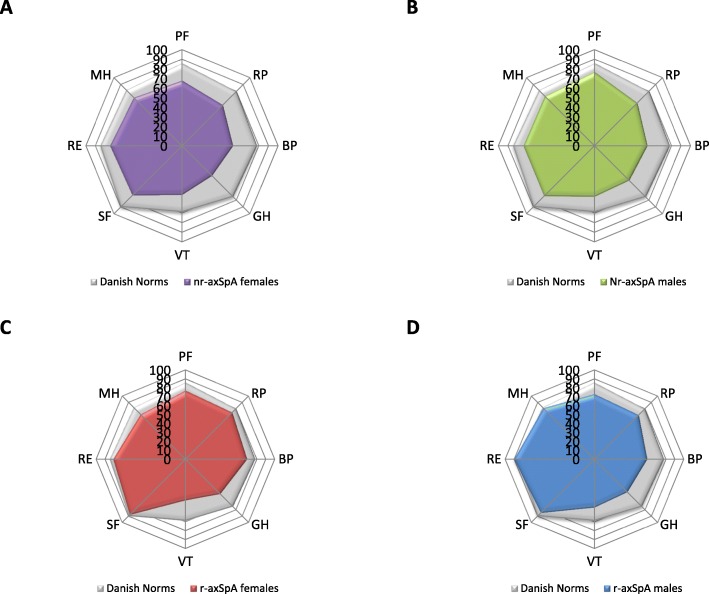
Mean SF-36 scores for Danish axSpA patients stratified on sex and axSpA classification. **a** nr-axSpA females. **b** nr-axSpA males. **c** r-axSpA females. **d** r-axSpA males. Mean SF-36 scores for Danish norms are also shown. PF, physical function; RP, role physical; BP, bodily pain; GH, general health; VT, vitality; SF, social functioning; RE, role emotional; MH, mental health

Visually, mean SF-36 subscale scores for our axSpA patients are illustrated in Fig. 3. Differences in HR-QoL across all domains compared to Danish normative values in this axSpA population are noticeable. The difference is most evident in nr-axSpA patients, with decrements in all eight domains. Moreover, HR-QoL is more impacted in nr-axSpA females compared with nr-axSpA males. In r-axSpA patients, the largest decrements are in BP, GH, and VT and only minor differences in SF and PF compared with Danish norms. r-axSpA did not appear to impact the SF, RE, and MH domains and was comparable to the general population.

## Discussion

This is to our knowledge the first extensive study performed comparing the clinical characteristics and PROMs in axSpA patients with an explicit objective regarding sex and type of clinical diagnosis. In line with prior studies, we found more men classified as r-axSpA compared with women (the male-to-female ratio was 3:1) and a female predominance in nr-axSpA patients [35]. We also found significantly shorter disease duration in nr-axSpA patients compared with r-axSpA patients [36]. When we compared patients with AS and nr-axSpA, HLA-B27 was not equally prevalent in patients with nr-axSpA and r-axSpA. The prevalence of HLA-B27 in our study reached 90% in r-axSpA patients and 57% in patients with nr-axSpA. The proportion of HLA-B27 positive in nr-axSpA patients was lower in our study compared to previous findings [37]. The low prevalence of HLA-B27 in nr-axSpA may reflect our eligibility criteria, as we used the ASAS “imaging arm” and thereby excluded the “clinical arm” in which HLA-B27 is mandatory. However, the proportion of axSpA patients classified by ASAS “clinical arm” is considered negligible in our axSpA population because the access to MRI was not limited.

Current treatment with NSAIDs and bDMARDs was similar between the subgroups of patients with axSpA, suggesting that patients were being treated similarly, regardless of the absence or presence of radiographic sacroiliitis. This emphasizes that the axSpA classification does not have a clinical importance.

Our study indicates that diffuse sensitization of the nociceptor system, as reflected by the number of tender points, has an impact on self-reported disease activity in patients classified as nr-axSpA. Awareness of concomitant diffuse sensitization of the nociceptor system (e.g., widespread pain) in axSpA patients is gaining importance, as it may cause poorer patient-reported outcome measurements and thereby a poorer treatment response [38–40]. We know from other studies that discordance between patient and physician assessment of disease activity is common in inflammatory rheumatic diseases and is associated with female sex [41, 42]. Whether this discordance is due to concomitant widespread pain syndrome is unclear. However, discordance between patient and physician assessment of disease activity challenges shared decision-making, and one can consider different thresholds for defining high disease activity (e.g., BASDAI) depending on sex and/or additionally try to better recognize the presence of widespread pain by assessing this condition more often and using better tools/questionnaires to recognize it especially but not exclusively in female patients in order to avoid over-treatment (especially with anti-inflammatory and immunosuppressants) for the wrong condition.

EAMs were frequent and, with the exception of a history of Achilles-enthesitis, equally prevalent in AS and nr-axSpA—sex did not have an impact on EAMs. The Charlson Comorbidity Index was lower than we expected and could be explained by the low median age in our axSpA patients. No differences in the CCI between patients classified as r-axSpA or nr-axSpA were found. However, male sex had a significant impact on the CCI. This could be explained by the fact that the majority of r-axSpA patients in our study are males, and r-axSpA patients have longer disease duration.

When exploring the impact of sex and axSpA classification on PROMs, our main finding was that being classified as nr-axspA had a negative impact on HR-QoL compared with patients classified as r-axSpA. All eight domains were lower compared with the Danish general population [43]. Furthermore, the impact was even more evident among nr-axSpA females. A possible explanation for this difference is that men and women appear to cope differently with the disease. The level of fatigue was higher among female nr-axSpA patients compared with the other subgroups, and it also contributes to negatively influencing HR-QoL [44]. Other studies have found that female gender is associated with higher fatigue levels, both in the general population and in axSpA patients [44, 45]. However, sex did not have a significant impact on fatigue in our study. Furthermore, we observed that SF-36 PCS scores were more affected compared to SF-36 MCS scores. This was noted in both nr-axSpa and r-axSpA patients. A recent meta-analysis reported a pooled mean SF-36 PCS score of 37.5 and SF-36 MCS score of 44.7 in r-axSpA patients [46]. Our results differed with higher scores for both the PCS and the MCS scores. A possible explanation for this observation could be that the inclusion of patients in our study was not restricted to disease activity or treatment, but consecutively enrolled in relation to routine visits at our outpatient clinics.

When exploring the impact of sex and axSpA classification on acute phase reactants, one main finding was that r-axSpA classification had an impact on CRP. In accordance with the present results, previous studies have demonstrated that r-axSpA patients have a higher CRP than nr-axSpA patients [47]. The median fecal calprotectin was not elevated in our axSpA patients. A possible explanation for this could be that any intake of NSAIDs was paused 14 days prior to the inclusion visit and due to the DMARD treatment. The evidence presented thus far supports the idea that systemic calprotectin plays a role in inflammatory rheumatic conditions, including axSpA [48]. Plasma calprotectin levels did not differ in nr-axSpA and r-axSpA patients in our study. This could indicate that activation of the innate immune cells plays an equal role in enthesitis and inflammation in both entities, as calprotectin triggers the innate immunity receptors resulting in inflammation [48, 49]. Currently, there is a lack of international consensus on which technique should be used to estimate calprotectin levels. This makes it difficult to compare studies.

The strengths of our study are the potential clinical implications. Our starting hypothesis was that the heterogenic clinical picture of axSpA might be further explored and elucidated by assigning participants to the subgroups based on the type of axSpA classification and sex. The limitation of this study includes the study design. A cross-sectional design was used, and therefore, no causality could be established. Conclusions can only be drawn based on the relationship between variables. To establish causality, it would be necessary to carry out longitudinal studies in order to evaluate over time the evolution of TPC and their relationship to self-reported disease activity or vice versa. However, since inclusion in this study was not restricted by disease severity or treatment, we believe our findings to be generalizable to the axSpA population.

## Conclusions

In summary, in this study, we observed that patients with r-axSpA are less affected on most PROMs, especially regarding generic HR-QoL measures when compared with axSpA patients not fulfilling the r-axSpA (i.e., AS) criteria. Moreover, HR-QoL is more negatively impacted in nr-axSpA females compared with nr-axSpA males. The number of tender points is associated with sex-related differences in self-reported disease activity in patients classified as nr-axSpA. Longitudinal studies are needed to explore the prevalence of other pain characteristics and their impact on clinical outcomes, PROMs, and treatment success rates in axSpA patients.

## Supplementary information

**Additional file 1.** Original protocol.

**Additional file 2.** Statistical analysis plan.

## Data Availability

The datasets used and analyzed during the present study are available from the corresponding author on reasonable request. The number to microarray data is not applicable.

## Abbreviations

AS: Ankylosing spondylitis
ASAS: Assessment of SpondyloArthritis international Society
ASDAS: Ankylosing Spondylitis Disease Activity Score
AxSpA: Axial spondyloarthritis
BASDAI: Bath Ankylosing Disease Activity Index
BASFI: Bath Ankylosing Spondylitis Functional Index
BASMI: Bath Ankylosing Spondylitis Metrology Index
CCI: Charlson Comorbidity Index
CRP: C-reactive protein
EAM: Extra-articular manifestation
GLM: General linear model
HR-QoL: Health-related quality of life
HLA-B27: Human leukocyte antigen B27
IQR: Interquartile range
MCS: Mental Component Summary
MRI: Magnetic resonance imaging
Nr: Non-radiographic
OMERACT: Outcome Measures in Rheumatology
PCS: Physical Component Score
PGA: Patient’s global assessment
PRP: Patient research partner
pSpA: Peripheral spondyloarthritis
PROMs: Patient-reported outcome measures
r-: Radiographic
SAP: Statistical analysis plan
SF-36: Short form 36 Health Survey
SpA: Spondyloarthritis
SPARCC: Spondyloarthritis Research Consortium of Canada
STROBE: Strengthening The Reporting of OBservational Studies in Epidemiology
TPC: Tender point count
VAS: Visual Analogue Scale
