# Supplementary material for: The impact of sex and disease classification on patient-reported outcome measures in axial spondyloarthritis: a descriptive prospective cross-sectional study
Source: Arthritis Res Ther. 2019 Oct 29;21:221. doi: 10.1186/s13075-019-2012-x (PMC6819525; doi:10.1186/s13075-019-2012-x)
Supplement: Supplementary file 1 — Additional file 1. Original protocol. [file 13075_2019_2012_MOESM1_ESM.pdf]

# Clinical characteristics and prevalence of extra-articular manifestations and co-morbidities in patients with spondylarthropathies: protocol for a prospective cross-sectional study from southern Denmark

**Rikke A. Andreasen[1,2], Robin Christensen[2], Lars E. Kristensen, Torkell Ellingsen[3], Inger Marie J. Hansen[1]**

**Author affiliations**

1: Department of Medicine, Svendborg Hospital, Odense University Hospital, Svendborg, Denmark.

2: Musculoskeletal Statistics Unit, The Parker Institute, Department of Rheumatology. Bispebjerg and Frederiksberg Hospital, The Capital Region of Copenhagen, Denmark.

3: Department of Rheumatology, Odense University Hospital, Odense, Denmark

**Correspondence to:**

Robin Christensen, MSc, PhD
Senior Biostatistician; Professor of Clinical Epidemiology
Head of Musculoskeletal Statistics Unit (MSU)
The Parker Institute, Department of Rheumatology.
Bispebjerg and Frederiksberg Hospital, The Capital Region of Copenhagen, Denmark.
Nordre Fasanvej 57
DK-2000 Copenhagen F
Denmark
e-mail: Robin.Christensen@regionh.dk
Tel: +45 3816 4165
Fax: +45 3816 4159

**Keywords:** spondylarthropathies, anterior uveitis, enthesitis, IBD, psorias, ankylosing spondylitis

**ABSTRACT**

Introduction**:** Spondyloarthritis (SpA) is a rheumatic disease with either predominantly axial inflammatory symptoms of the spine and sacroiliac joints or predominantly peripheral arthritis. Extra-articular manifestations are associated with SpA. The most common manifestations are anterior uveitis, psoriasis and inflammatory bowel disease (IBD). Also cardiovascular disease (CVD) is frequently seen in patients with SpA, which is partly explained by systemic inflammatory activity, but the usual cardiovascular risk factors contribute as well. Peripheral arthritis occurs in approximately 30% of the patients, and shows an asymmetrical, oligoarticular pattern. Other common joint complaints are due to enthesitis, which manifest as extra-articular bony tenderness in areas such as the Achilles tendon, iliac crests and plantar fascia. Although less common, organ involvement in SpA can also be located in the heart, lungs, skin and kidneys. Overall it is important to realize that extra-articular manifestations frequently occur in patients with SpA and should be taken into account in the choice of treatment.

**Method and analysis:**

We anticipate that we will be able to enroll 100 adult individuals (≥ 18 years of age) diagnosed with SpA. Among these individuals we want to explore the prevalence of concomitant extra articular manifestations. Data (duration of illness, diagnostic category, disease activity, extra-articular manifestations, blood, urine, and faeces samples, patient reported outcomes and comorbidity) will be collected from each participant. For all the clinical characteristics we will explore whether any of these have an impact on the reported disease activity according to the international standards – the Bath Ankylosing Spondylitis Disease Activity Index

**Ethics and dissemination:**

For the practitioner it is important to know whether certain co-morbidities have an impact on the disease activity, and subsequently to explore whether any of these could have prognostic value in a longitudinal study setting. The study will be approved by the ethics committee for the Region of Southern Denmark and the Data protection at Region of Southern Denmark. Dissemination will occur through presentations and publication in international peer-reviewed journals.

## INTRODUCTION

Spondyloarthopathies (SpA) is a heterogenous group of chronic rheumatic diseases with overlapping symptoms including psoriatic arthritis (PsA), ankylosing spondylitis (AS) arthritis associated with inflammatory bowel disease (enteropathic arthritis), reactive arthritis and undifferentiated SpA (1). SpA can be dominated by peripheral joint involvement, classified as peripheral SpA, or by inflammatory back pain, classified as axial SpA (ax-SpA). Furthermore ax-SpA is subdivided into two groups: Nonradiographic and radiographic. The Assessment of Spondyloarthritis International Society (ASAS) has made a set of criteria for recognizing patients with early ax-SpA to optimize treatment and to reduce the risk of irreversible joint damage. These criteria are summarized in **Figure 1**(2). It is estimated that peripheral arthritis occurs in around one third of the patients diagnosed with SpA, especially the large joints on the lower limbs are involved, often in an asymmetrical oligoarticular pattern (3). Furthermore enthesitis is a common manifestation in SpA. Next to the spinal and articular symptoms, many patients with SpA also have extra-articular manifestations which contribute to reduced quality of life (4). Recently, nephrolithiasis was identified as a common (up to 10%) and unrecognized extra-articular manifestation in AS (5).

The etiology of SpA is complex and not fully understood. It is known that SpA is associated with multiple genes, such as HLA-B27 (6). However, the pathogenesis of SpA remains largely unknown. The complexity of the disorder indicates a multifactorial etiology involving multiple biological processes or pathways (7). Initially, SpA was considered a disease primarily of the male gender, but the profile has changed over time, and SpA has been regognized among females as well (8-10). Although specific cardiac manifestations as aortic valve disease or conduction disturbances is associated with SpA (especially AS), accelerated atherosclerosis also renders these patients more at risk cardiovascular disease. The cardiovascular risk factors and the systemic inflammation contribute to cardiovasular events (11, 12). Fecal calprotectin is a protein in neutrophil granulocytes and macrophages and is first found and described in 1980 (13) More and more studies focus on fecal calprotectin in IBD and confirm its value in

diagnosis, disease activity evaluation, effect evaluation, and relapse monitor (14) A prospective cohort study demonstrates that the sensitivity and specificity of CD are 100% and 97% (cutoff 30 mg/L) when compared to irritable bowel syndrome (IBS) (15) A strong link between AS and IBD is suggested. Recent studies found that 6.5% of patients with AS will develop IBD, and the prevalence of AS in patients with IBD is up to 10 % (16) Furthermore, even without clinical symptoms, up to 60 % of the patients with AS present gut inflammation at colonoscopy (17). Awareness of extra-articular manifestations among clinicians is important in view of their role in the diagnostic process, for treatment choices and for health related quality of life. The treatment of SpA aims to control the systemic inflammation and halt the progression of joint destruction and disability, and early treatment is important. Medical treatment in SpA spans from non steroidal anti inflammatory drug (NSAID), intraarticular corticosteroid injection to systemic treatment with synthetic Disease Modifying Anti-rheumatic drugs (DMARD) or biological treatment in terms of tumor necrosis factor inhibitors (TNFi) (3, 18)

**Rationale and hypothesis**

In AS first line of therapy is NSAID, but in patients with simultaneous IBD these should be administered with caution as some studies have suggested that they exacerbate intestinal inflammation (19). Early induction of an anti-TNF therapy among these patients is recommended(20). Furthermore enthesitis is a typical feature in SpA and the treatment of these manifestations seem to be challenging as well. Studies have showed that anti-TNF therapy is superior to conventional DMARD in patients with longstanding enthesitis (21, 22). Uveitis is an acute inflammation of the uvea and is a common and potential serious extra-articular manifestation in patients with SpA. In most cases acute uveitis can be successfully treated by the opthalmologists with local corticosteroids, but in some cases it develops into a refractory uveitis, and treatment with TNFi is recommended (as azathioprine and methotrexate do not have sufficient effect on the disease activity of SpA) (23, 24).

We anticipate that extra-articulare manifestations among patients with SpA is

underestimated and therefore undertreated, and contributes to a reduced quality of life. Spondyloarthritic disorders are generally diagnosed more often in men than in women (25). In reference to the most representative form of these disorders, ankylosing spondylitis, three male cases are documented for every female case (25). However, since the introduction of the new ASAS criteria for axial spondyloarthropathies these differences are no longer so apparent (26). SpA appears to be more frequent in men than in women, particularly in its axial presentations (27). Nevertheless, little is known of the differential clinical expression of SpA between males and females. The explanation for these differences is not clear, and the participation of multifactorial parameters cannot be discarded. A few studies have analysed gender as a prognostic factor of the SpA (8-10) and there is a need to determine the particularities and severity of SpA in the female gender. We hypothesise that the amount of self-reported disease activity is more pronounced in woman than in men. (i.e. Bath Ankylosing Spondylitis Disease Activity Index (BASDAI (28)) and Bath Ankylosing Spondylitis Functional Index (BASFI (29)). As it seems to be a link between IBD and SpA, we also aimed to measure faecal calprotectin in patients with any disease within the SpA spectrum.

**Objectives**

The overall objective of this cross-sectional study is to perform a characterization of SpA patients, to identify clinical phenotypes of SpA and ultimately elucidate factors predictive of disease outcome. This insight will be important for rheumatologists in the perspective of making individualized treatment strategies, prognostic stratification and patient counseling.

**Aims**

1. To analyze the distribution according to specific concomitant conditions in the SpA group:
   Ankylosing spondylitis was diagnosed when the patients fulfilled the modified New York criteria (30), psoriatic arthritis was diagnosed when patients fulfilled the CASPAR criteria (31), enteropathic arthritis was diagnosed when patients exhibited inflammatory axial and/or peripheral

joint involvement associated to a confirmed IBD (Crohn's disease or ulcerative colitis), SpA was diagnosed when patients fulfilled the ASAS criteria (2), reactive arthritis was diagnosed when an asymmetric inflammatory oligoarthritis and/or inflammatory low back pain associated to confirmed genitourinary or gastrointestinal tract.

2. To characterize the extra-articular manifestations according to ASAS criteria, however also including the recently described nephrolithiasis among SpA patients:
   Anterior uveitis, (past or present), inflammatory bowel disease (past or present) enthesitis (past or present), psoriasis (past or present) dactylitis (past or present) and furthermore to assess other features as: urethritis, pyoderma gangrenosum, keratoderma blennorrhagica, erythema nodosum (past or present), nephrolithiasis (past or present), and urine components.

3. To estimate the male-to-female ratio among patients with SpA

4. To investigate faecal calcprotectin in patients with any disease in the SpA spectrum

5. To analyze the influence of gender on self-reported disease activity:
   Estimation of disease activity and functional status is evaluated using BASDAI, BASFI and 36-Item Short-Form Health Survey (SF-36)

6. To analyze co-morbidity among SpA patients using Charlson Comorbidity Index

A priori hypotheses are as follows:

A. The amount of self-reported disease activity is more pronounced in woman than in men

B. Females are more likely to have peripheral joint involvement rather than axial compared to men
C. Extra articular manifestations are more frequent seen in males compared with females
D. Faecal calcprotectin is higher in men than women
E. Urine electrolytes are altered and related to nephrolithiasis and NSAID exposure

## METHODS

### Study design

The study will be designed as a cross-sectional cohort design with prospective enrolment of patients with SpA over time. Information about the patients and their exposures will be collected at a single center at one visit (figure 2) according to the clinical standards in Denmark. Examinations will be carried out on the same day. Participant inclusion is expected to begin during 2016.

### Participants

Patients with any disease of the SpA spectrum seen in the Department of Rheumatology, Odense University Hospital, Svendborg/Odense will be recruited. To be considered for inclusion, participants must have the ability and willingness to give written informed consent and to meet the requirements of this protocol. Participants must be at least 18 years of age. Participants will be excluded from the study if any of the following criteria are present: (1) no consent, (2) does not understand Danish, (3) age < 18 years.

All subjects will be informed about the project's purpose and content both oral and written - as per the Research Ethics Committee. Participants will receive information by MD Rikke Asmussen Andreasen. Patients are made aware of the possibility to bring one accompanying person. The interview will take place in an undisturbed space at the rheumatology clinic. The subjects were informed that they could at any time withdraw their commitment to participate in the project, without this will affect the current or subsequent treatment or control. The subjects give permission to the investigator to have direct access to source data / documents (including DANBIO) for the monitoring, auditing and / or inspection

by the Research Ethics Committee or the Data Protection Agency. On the consent form, the investigator signing that written and oral information is given as above.

**Variables and outcome measures**

Included patients will undergo an examination programme to collect variables shown in **table 1**.

Informations on: (1) demographic data (age, sex, height, weight, educational level), (2) clinical characteristics (symptom duration, duration since SpA diagnosis, and type of the form of clinical involvement, HLA-B27, family history and extra-articular manifestations (3) activity data and monitoring methods (erythrocyte sedimentation rate (ESR), CRP, visual analogue scale (VAS) for pain and acivity, morning stiffness, joint assessment, painful enthesis [BASDAI]), (4) employment status and functional ability [BASFI]) and SF-36 (5) comorbidity will be obtained by interview.

The BASFI is a 10-item scale on which respondents rate the degree of difficulty they have in performing certain tasks, using visual analogue scales (VAS) from 0 (easy) to 100 (impossible). The mean of the 10 responses is the BASFI score. The BASDAI is a 6-item scale to measure severity of fatigue, spinal and peripheral joint pain, localized tenderness and morning stiffness, using the VAS scale as well. The Medical Outcomes Study SF-36 is a generic health status questionnaire that was developed as a tool to compare various aspects of health status across a general and broad patient population (32-34). The SF-36 examines eight general health domains: physical functioning, role limitations due to physical health problems, bodily pain, general health perceptions, vitality, social functioning, role limitations due to emotional problems and mental health. Furthermore, a physical and mental component summary score can be calculated. We will use the Danish version of SF-36 (35) which uses a 4-week recall period. For Peripheral articular involvement 44 tender/swollen joint count will be assessed.

**Table 1** Summary of measures to be collected

| | |
|---|---|
| **Demographics** | X |
| Sex (M/F) | X |
| Age (year) | X |
| Height (cm) | X |
| Weight (Kg) | X |
| BMI (Kg/m$^2$) | X |
| **Disease Characteristics** | |
| Symptom duration (months) | X |
| Axial disease (yes/no) | X |
| MRI positive (yes/no) | |
| Peripheral joint involvement (yes/no) | X |
| **Medication** | |
| Prior NSAID (yes/no) | X |
| Current NSAID at assessment (yes/no) | X |
| Prior DMARD therapy (yes/no) | X |
| MTX dose (mg/week) | X |
| Other current DMARD therapy | X |
| No. of previous biologics used (if any) | X |
| Name of current biological agent | X |
| Dose of prednisolone orally at assessment (mg/week) | X |
| Intramuscular glucocorticoid injections in the previous 3 month (mg) | X |
| **Clinical examination** | |
| 46 swollen joint count | X |
| 46 tender joint count | X |
| BASMI | X |
| **SpA features** | |
| Anterior uveitis, past or present (yes/no) | X |
| Inflammatory bowel disease, past og present (yes/no) | X |
| Psoriasis, past or present (yes/no) | X |
| Preceding infection (yes/no) | X |
| Total enthesitis count | X |
| Dactylitis count | X |

| | |
|---|---|
| **Co-morbidities** | |
| Charlson Comorbidity Index | X |
| **Urine sample** | X |
| Basic metabolic screening | X |
| S-creatinin | X |
| S-ioniseret calcium | X |
| S-urat | X |
| Urine-pH (dip-stick) | X |
| Urine Calcium | X |
| Urine Chloride | X |
| Urine Potassium | X |
| Urine Sodium | X |
| Urine Creatinin | X |
| Urine albumin | X |
| Urine Oxalate | X |
| **Patient-reported outcomes** | |
| BASDAI | X |
| BASFI | X |
| SF-36 | X |
| **Blood samples** | |
| *HLA-B27 (positive/negative) | X |
| **CRP, ESR, vitamin D, immunoglobulin A, ALAT, alkaline phosphatise, creatinine, estimated GFR, haemoglobin, erythrocyte volume fraction, MCHC, MCV, leucocytes, differential count, thrombocytes, P-calprotectin | X |
| **Stool sample** | |
| Faecal calprotectin (mg/L) | X |

*will not be repeated if already taken

**will not be repeated if taken within the past 2 weeks

CRP, C-reactive protein; ESR, erythrocyte sedimentation rate; ALAT, alanine transaminase; GFR, glomerular filtration rate; MCHC, mean corpuscular

haemoglobin concentration; MCV, mean corpuscular volume; MTX; methotrexate, DMARD; disease-modifying antirheumatic drugs

**Clinical examination and blood samples**

Blood samples as specified in table 1 will be collected by a trained laboratory technician and treated according to set procedures.

**Sample size considerations and statistical analyses**

This study is designed as an exploratory study. It is anticipated that 100 participants are likely to be included during a period of 6 months. A sample size of 100 individuals is considered sufficient to test the hypothesis: 'is there a difference between the reported BASDAI in SpA patients whether they are men or women? For a two-sample pooled t test of a normal mean difference with a two-sidedsignificance level of 0.05, assuming a common standard deviation of 1.5 BASDAI units, a total sample size of 100 assuming a balanced design (1:1 male-female ratio) has a sufficient power (91%) to detect a mean difference of 1 BASDAI units.

All descriptive statistics and test will be reported in accordance with the recommendations of the "Enhancing the QUAlity and Transparency Of health Research" (EQUATOR) network (36): the STROBE Statement (37). We consider p values less than 0.05 (and 95% confidence intervals excluding the null) to be statistically significant.

**DISCUSSION, ETHICS AND DISSEMINATION**

We will apply the ethics committee for the region of Southern Denmark. Dissemination will occur through presentation and publication in international peer-reviewed journals.

Knowledge about the presence of extra-articular manifestations in an individual may be useful for rheumatologists to optimize the treatment.

This study is an attempt to evaluate the characteristics of the patients within the SpA spectrum regarding extra-articular manifestations, disease onset (age of onset, age of diagnosis, delay in diagnosis, and first symptom at presentation) clinical activity evaluated by ESR, CRP, BASDAI and BASFI. It is our hope that the

results of this study may add to the knowledge of the disease pattern among patients diagnosed with SpA.

**Figure 1** ASAS Classification Criteria for Axial Spondyloarthritis (SpA)

| | | |
|---|---|---|
| Sacroilitis on imaging* | OR | HLA-B27 |
| Plus | | Plus |
| ≥ 1 SpA feature | | ≥ 2 other SpA features |

*active inflammation on MRI highly suggestive of sacroilitis associated with SpA, or definite radiographic sacroilitis according to the modified New York criteria

**SpA features:**
Inflammatory back pain
Arthritis
Enthesitis
Uveitis
Dactylitis
Psoriasis
IBD*
Family history for SpA
HLA-B27
Elevated CRP
Good reponse to NSAID

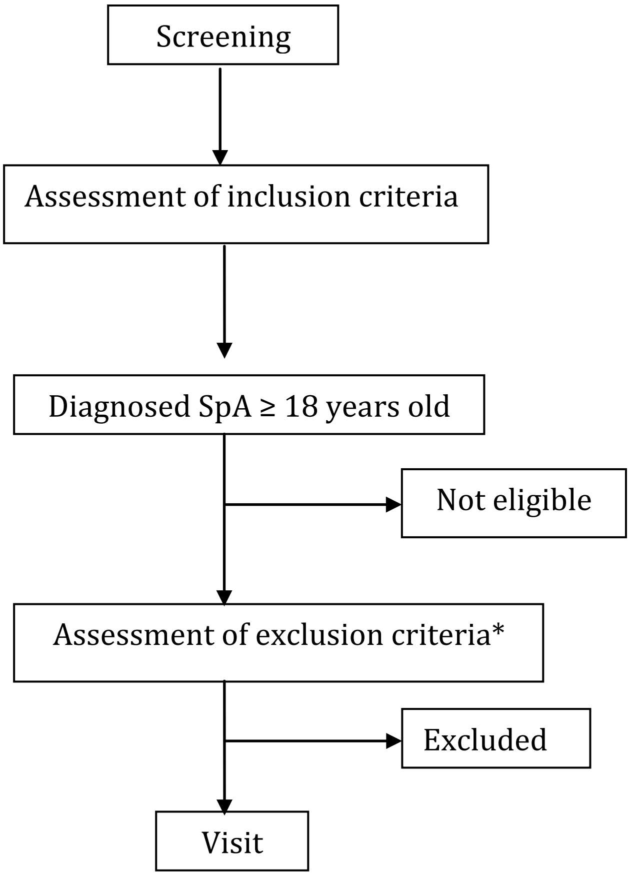


**Figure 2** Overview of participant flow

*Exclusion criteria: (1) No consent, (2) Does not understand Danish, (3) Age < 18 years

**Contributors:** Rikke A. Andreasen serves as the principal investigator for this study. Robin Christensen, Lars E. Kristensen, Torkell Ellingsen and Inger Marie J. Hansen participated in the design of the study and drafting of the protocol

**Funding** The initiative for the project was taken by The Department of Medicine, Section of Rheumatology, Odense University Hospital, Svendborg. The project is funded by a PhD stipend from the PhD fund of Region Southern Denmark. The amount is 500.000 DKR and is covering 1 year salary (including 1 year study fee) to the PhD student. The investigator has no affiliation to the PhD fund of Region Southern Denmark. Additional funds will be applied. There will be no fees for the participants.

**Competing interests** None.

**Ethics approval** Will be approved by the southern region of Denmark's Ethics Committee and the Data protection at the Region of Southern Denmark.

**REFERENCES**


1. van der Horst-Bruinsma IE, Nurmohamed MT. Management and evaluation of extra-articular manifestations in spondyloarthritis. Therapeutic advances in musculoskeletal disease. 2012;4(6):413-22.
2. Molto A, Paternotte S, van der Heijde D, Claudepierre P, Rudwaleit M, Dougados M. Evaluation of the validity of the different arms of the ASAS set of criteria for axial spondyloarthritis and description of the different imaging abnormalities suggestive of spondyloarthritis: data from the DESIR cohort. Annals of the rheumatic diseases. 2014.
3. Wilson G, Folzenlogen DD. Spondyloarthropathies: new directions in etiopathogenesis, diagnosis and treatment. Missouri medicine. 2012;109(1):69-74.
4. Braun J, Kiltz U, Baraliakos X, van der Heijde D. Optimisation of rheumatology assessments - the actual situation in axial spondyloarthritis including ankylosing spondylitis. Clinical and experimental rheumatology. 2014;32(5 Suppl 85):S-96-104.
5. Jakobsen AK, Jacobsson LT, Patschan O, Askling J, Kristensen LE. Is nephrolithiasis an unrecognized extra-articular manifestation in ankylosing spondylitis? A prospective population-based Swedish national cohort study with matched general population comparator subjects. PloS one. 2014;9(11):e113602.
6. Zhao J, Chen J, Yang TH, Holme P. Insights into the pathogenesis of axial spondyloarthropathy from network and pathway analysis. BMC systems biology. 2012;6 Suppl 1:S4.
7. Olivieri I, van Tubergen A, Salvarani C, van der Linden S. Seronegative spondyloarthritides. Best practice & research Clinical rheumatology. 2002;16(5):723-39.
8. Roussou E, Sultana S. Spondyloarthritis in women: differences in disease onset, clinical presentation, and Bath Ankylosing Spondylitis Disease Activity and Functional indices (BASDAI and BASFI) between men and women with spondyloarthritides. Clinical rheumatology. 2011;30(1):121-7.
9. Gran JT, Ostensen M, Husby G. A clinical comparison between males and females with ankylosing spondylitis. The Journal of rheumatology. 1985;12(1):126-9.
10. de Carvalho HM, Bortoluzzo AB, Goncalves CR, da Silva JA, Ximenes AC, Bertolo MB, et al. Gender characterization in a large series of Brazilian patients with spondyloarthritis. Clinical rheumatology. 2012;31(4):687-95.
11. Szabo SM, Levy AR, Rao SR, Kirbach SE, Lacaille D, Cifaldi M, et al. Increased risk of cardiovascular and cerebrovascular diseases in individuals with ankylosing spondylitis: a population-based study. Arthritis and rheumatism. 2011;63(11):3294-304.
12. Mathieu S, Pereira B, Soubrier M. Cardiovascular events in ankylosing spondylitis: An updated meta-analysis. Seminars in arthritis and rheumatism. 2014.
13. Roseth AG, Fagerhol MK, Aadland E, Schjonsby H. Assessment of the neutrophil dominating protein calprotectin in feces. A methodologic study. Scandinavian journal of gastroenterology. 1992;27(9):793-8.

14. Smith LA, Gaya DR. Utility of faecal calprotectin analysis in adult inflammatory bowel disease. World journal of gastroenterology : WJG. 2012;18(46):6782-9.
15. Tibble J, Teahon K, Thjodleifsson B, Roseth A, Sigthorsson G, Bridger S, et al. A simple method for assessing intestinal inflammation in Crohn's disease. Gut. 2000;47(4):506-13.
16. Stolwijk C, Essers I, van Tubergen A, Boonen A, Bazelier MT, De Bruin ML, et al. The epidemiology of extra-articular manifestations in ankylosing spondylitis: a population-based matched cohort study. Annals of the rheumatic diseases. 2014.
17. Van Praet L, Jans L, Carron P, Jacques P, Glorieus E, Colman R, et al. Degree of bone marrow oedema in sacroiliac joints of patients with axial spondyloarthritis is linked to gut inflammation and male sex: results from the GIANT cohort. Annals of the rheumatic diseases. 2014;73(6):1186-9.
18. Molto A, Paternotte S, Claudepierre P, Breban M, Dougados M. Effectiveness of tumor necrosis factor alpha blockers in early axial spondyloarthritis: data from the DESIR cohort. Arthritis & rheumatology (Hoboken, NJ). 2014;66(7):1734-44.
19. Kefalakes H, Stylianides TJ, Amanakis G, Kolios G. Exacerbation of inflammatory bowel diseases associated with the use of nonsteroidal anti-inflammatory drugs: myth or reality? European journal of clinical pharmacology. 2009;65(10):963-70.
20. Van den Bosch F, Kruithof E, De Vos M, De Keyser F, Mielants H. Crohn's disease associated with spondyloarthropathy: effect of TNF-alpha blockade with infliximab on articular symptoms. Lancet. 2000;356(9244):1821-2.
21. Tse SM, Laxer RM, Babyn PS, Doria AS. Radiologic Improvement of juvenile idiopathic arthritis-enthesitis-related arthritis following anti-tumor necrosis factor-alpha blockade with etanercept. The Journal of rheumatology. 2006;33(6):1186-8.
22. Milia AF, Ibba-Manneschi L, Manetti M, Benelli G, Generini S, Messerini L, et al. Evidence for the prevention of enthesitis in HLA-B27/hbeta(2)m transgenic rats treated with a monoclonal antibody against TNF-alpha. Journal of cellular and molecular medicine. 2011;15(2):270-9.
23. Fotiadou C, Lazaridou E, Kemanetzi C, Kyrmanidou E, Ioannides D. Recalcitrant psoriatic uveitis and anti-tumor necrosis factor-alpha monoclonal antibodies: experience from a psoriasis referral center. International journal of dermatology. 2014.
24. Pham T, Fautrel B, Dernis E, Goupille P, Guillemin F, Le Loet X, et al. Recommendations of the French Society for Rheumatology regarding TNFalpha antagonist therapy in patients with ankylosing spondylitis or psoriatic arthritis: 2007 update. Joint, bone, spine : revue du rhumatisme. 2007;74(6):638-46.
25. Zochling J, Smith EU. Seronegative spondyloarthritis. Best practice & research Clinical rheumatology. 2010;24(6):747-56.
26. Rudwaleit M. New approaches to diagnosis and classification of axial and peripheral spondyloarthritis. Current opinion in rheumatology. 2010;22(4):375-80.
27. Queiro R, Sarasqueta C, Torre JC, Tinture T, Lopez-Lagunas I. Spectrum of psoriatic spondyloarthropathy in a cohort of 100 Spanish patients. Annals of the rheumatic diseases. 2002;61(9):857-8.

28. Garrett S, Jenkinson T, Kennedy LG, Whitelock H, Gaisford P, Calin A. A new approach to defining disease status in ankylosing spondylitis: the Bath Ankylosing Spondylitis Disease Activity Index. The Journal of rheumatology. 1994;21(12):2286-91.
29. Calin A, Garrett S, Whitelock H, Kennedy LG, O'Hea J, Mallorie P, et al. A new approach to defining functional ability in ankylosing spondylitis: the development of the Bath Ankylosing Spondylitis Functional Index. The Journal of rheumatology. 1994;21(12):2281-5.
30. van der Linden S, Valkenburg HA, Cats A. Evaluation of diagnostic criteria for ankylosing spondylitis. A proposal for modification of the New York criteria. Arthritis and rheumatism. 1984;27(4):361-8.
31. Taylor W, Gladman D, Helliwell P, Marchesoni A, Mease P, Mielants H. Classification criteria for psoriatic arthritis: development of new criteria from a large international study. Arthritis and rheumatism. 2006;54(8):2665-73.
32. McHorney CA, Ware JE, Jr., Lu JF, Sherbourne CD. The MOS 36-item Short-Form Health Survey (SF-36): III. Tests of data quality, scaling assumptions, and reliability across diverse patient groups. Medical care. 1994;32(1):40-66.
33. McHorney CA, Ware JE, Jr., Raczek AE. The MOS 36-Item Short-Form Health Survey (SF-36): II. Psychometric and clinical tests of validity in measuring physical and mental health constructs. Medical care. 1993;31(3):247-63.
34. Ware JE, Jr., Sherbourne CD. The MOS 36-item short-form health survey (SF-36). I. Conceptual framework and item selection. Medical care. 1992;30(6):473-83.
35. Bjorner JB, Thunedborg K, Kristensen TS, Modvig J, Bech P. The Danish SF-36 Health Survey: translation and preliminary validity studies. Journal of clinical epidemiology. 1998;51(11):991-9.
36. Christensen R, Bliddal H, Henriksen M. Enhancing the reporting and transparency of rheumatology research: a guide to reporting guidelines. Arthritis research & therapy. 2013;15(1):109.
37. Vandenbroucke JP, von Elm E, Altman DG, Gotzsche PC, Mulrow CD, Pocock SJ, et al. Strengthening the Reporting of Observational Studies in Epidemiology (STROBE): Explanation and elaboration. International journal of surgery (London, England). 2014;12(12):1500-24.
