# Supplementary material for: The impact of sex and disease classification on patient-reported outcome measures in axial spondyloarthritis: a descriptive prospective cross-sectional study
Source: Arthritis Res Ther. 2019 Oct 29;21:221. doi: 10.1186/s13075-019-2012-x (PMC6819525; doi:10.1186/s13075-019-2012-x)
Supplement: Supplementary file 2 — Additional file 2. Statistical analysis plan. [file 13075_2019_2012_MOESM2_ESM.pdf]

Clinical characteristics and patient-reported outcome measures in patients with axial spondyloarthritis:

Statistical Analysis Plan for a prospective cross-sectional study from southern Denmark

SAP Version 2.1, October 30, 2018

Collaborators

Rikke Asmussen Andreasen, Lars Erik Kristensen, Kenneth Egstrup, Xenofon Baraliakos, Vibeke Strand, Hans Christian Horn, Inger Marie Jensen Hansen, Robin Christensen*, Torkell Ellingsen*.

Trial registration
The study was approved by the Region of Southern Denmark's Ethics committee (S-20150219) and the study protocol is available from www.Parkerinst.dk.

Protocol version

This document has been written based on information contained in the study protocol version 3, dated 20 December 2015.

Statistical analyst
Rikke Asmussen Andreasen, MD,
[1]Department of Medicine, Section of Rheumatology, Odense University Hospital, Svendborg
DK-5700 Svendborg, Denmark.
[2]Musculoskeletal Statistics Unit, The Parker Institute, Bispebjerg and Frederiksberg Hospital,
DK-2000 Copenhagen F, Denmark.

Statistical advisor
[1,2]Robin Christensen, MSc, PhD; Biostatistician
Professor of Biostatistics and Clinical Epidemiology,
[1]Head of Musculoskeletal Statistics Unit, The Parker Institute, Bispebjerg and Frederiksberg Hospital,
DK-2000 Copenhagen F, Denmark.
[2]Department of Rheumatology, Odense University Hospital,
DK-5000 Odense, Denmark.

Purpose of the Statistical Analysis Plan

The SAP was initiated after the study protocol was registered and published online (www.Parkerinst.dk), but prior to reviewing data or conducting final analyses. The purpose of the SAP is to give a more transparent and detailed elaboration on the statistical analyses of the variables prospectively collected. Deviations from the pre-specified protocol are explained in the SAP. The reporting of the study will follow the *Strengthening the Reporting of Observational Studies in Epidemiology* (STROBE) (1).

Background and rationale

Spondyloarthritis (SpA) is a heterogenous group of chronic rheumatic diseases; it can be dominated by peripheral joint involvement, classified as peripheral SpA (pSpA), or by inflammatory back pain, classified as axial SpA (axSpA). Furthermore axSpA is subdivided into two stages: Nonradiographic (nr-axSpA) and radiographic (r-axSpA, formerly known as ankylosing spondylitis, AS). Next to the spinal and articular symptoms, many patients with axSpA also have extra-articular manifestations (EAMs; e.g. uveitis, enthesitis, psoriasis, inflammatory bowel disease) which contribute to reduced health-related quality of life (2). We anticipate that EAMs among patients with axSpA are underestimated and therefore undertreated. Besides EAMs, axSpA patients have a higher risk of comorbidities, including diabetes mellitus, renal disease, cardiovascular disease (CVD), and pulmonary disease (3-5). Awareness of comorbidities among clinicians is important in view of their role for treatment choices for axSpA patients. Other medical specialists and care professionals do of course also have their place. AxSpA disorders are generally diagnosed more often in men than in women (6). In reference to the most representative form of these disorders, r-axSpA, three male cases are documented for every female case (6). In contrast to r-axSpA, nr-axSpA patients show little difference in prevalence among males and females (7). Nevertheless, little is known of the differential clinical expression of axSpA between males and females, and females are often underrepresented in clinical research (8, 9). The higher incidence of r-axSpA in males compared to females has stimulated research on gender differences in axSpA. Few studies have analysed gender as a prognostic factor for course of disease in axSpA patients (8, 10, 11) and there is a need to determine the particularities and severity of axSpA in the female gender.

Aims

The overall objective of this cross-sectional study is to present a characterisation of axSpA patients, to

illuminate patient-reported outcome measures, and ultimately elucidate factors associated with disease outcome.

Hypotheses

We hypothesise that the amount of self-reported disease activity is more pronounced in women than in men (i.e. Bath Ankylosing Spondylitis Disease Activity Index [BASDAI (12)], and Bath Ankylosing Spondylitis Functional Index [BASFI (13)]).

Objectives

The primary objective of this study is to analyse the impact of gender on self-reported disease activity in axSpA patients based on the type of clinical diagnosis.

Secondary objectives are:
a. To determine the relationship between the continuous measure of muscular tenderpoints (TP (14)) and the self-reported disease activity (i.e. BASDAI).

b. To determine the prevalence of EAMs and comorbidities, as defined by the Charlson comobidy index, in axSpA patients.

c. To compare the impact on health-related quality of life of axSpA patients, specifically in terms of how axSpA classification differentially affects aspects of physical, mental and social wellbeing.

Sample size considerations

This study was designed as an exploratory study. A sample size of 100 individuals was considered sufficient to test the hypothesis: 'is there a difference between reported BASDAI scores in axSpA patients by men vs women? For a two-sample pooled t test of a normal mean difference with a two-sided significance level of 0.05, assuming a common standard deviation of 1.5 BASDAI units, a total sample size of 100 assuming a balanced design (1:1 male-female ratio) has sufficient power (91%) to detect a mean difference of 1 BASDAI units. All descriptive statistics and tests will be reported in accordance with the recommendations of the

"Enhancing the QUAlity and Transparency Of health Research" (EQUATOR) network (15): the STROBE Statement (1). We consider p values less than 0.05 (and 95% confidence intervals excluding the null) to be statistically significant.

Statistical Analysis Plan (SAP)

Study design

The study is designed as a cross-sectional study with prospective enrolment of axSpA patients. Information about the patients and their exposures were collected at a single centre at one visit according to the clinical standards in Denmark. Examinations were conducted on the same day. The inclusion period was between 1st of April 2016 and 30th of March 2018.

Study participants

Patients with axSpA seen in the Department of Rheumatology, Odense University Hospital, Svendborg/Odense were recruited. We used the ASAS classification criteria for axSpA (imaging arm) (16), and the modified New York criteria for r-axSpA (17). To be considered for inclusion, participants must have the ability and willingness to give written informed consent and to meet the requirements of the pre-specified protocol. Participants were excluded from the study if any of the following criteria were present: (1) no consent, (2) does not understand Danish, (3) age < 18 years.

Statistical interim analyses and stopping guidance

Preliminary results have been presented at the EULAR congress in 2017 in Madrid (18). 80 participants were included at that time point.

Timing of final analysis

The first main report/publication of the trial will be prepared when the SAP is completed, and the research team has approved it (anticipated to be November 2018).

Urine samples were collected in this study to analyse whether the urine electrolytes were altered and related to nephrolithiasis in axSpA patients. The results will not be part of the proposed manuscript, but will be presented in a separate future paper.

Confidence intervals and P values

Level of statistical significance and use of confidence intervals: All applicable statistical tests will be 2-sided and will be performed using a 5% statistical significance level. No correction for multiple testing will be conducted; instead the results will be interpreted with caution in the context multiplicity.

Statistical Analysis Plan (SAP)

Protocol deviations

To specify the aim of this study and to clarify the statistical analyses, some deviations from the pre-specified protocol were made.

Aims:

Protocol: The overall objective of this cross-sectional study is to perform a characterisation of SpA patients, to identify clinical phenotypes of SpA and ultimately elucidate factors predictive of disease outcome.

SAP: The overall objective of this cross-sectional study is to present a characterisation of axSpA patients, to illuminate patient-reported outcome measures, and ultimately elucidate factors associated with disease outcome.

Rationale: Spondyloarthritis (SpA) is a heterogeneous group of chronic rheumatic diseases with overlapping symptoms including psoriatic arthritis (PsA), r-axSpA, arthritis associated with inflammatory bowel disease (enteropathic arthritis) and undifferentiated SpA . SpA can be dominated by peripheral joint involvement, classified as peripheral SpA (pSpA), or by inflammatory back pain, classified as axial SpA (ax-SpA).

Eligibility

The number of ineligible patients will be reported with reasons for ineligibility.

Recruitment

A flowchart template modified from http://www.consort-statement.org comprising the number of people screened, eligible, consented, and included in the analyses will be used (**Figure 1**).

Collected variables

The following information was collected at the study visit (**Table 1)**. Information on: (1) demographic, (2) disease characteristics, (3) medication, (4) axSpA features, (5) patient reported outcome measures (PROMs) (6) co-morbidities using Charlson Co-morbidity Index (CCI), (19). An experienced physician (RAA) performed a clinical examination and performed a 44 tender/swollen joint count, the Bath Ankylosing Spondylitis Metrology Index (BASMI), an 18-sites tender point count, and an enthesitis score. The core domains set recommended by the ASAS/OMERACT: physical function, pain, spinal mobility, patient's global

assessment (PGA), peripheral joints/enthuses, spinal stiffness, acute phase reactants and fatigue were assessed using the instruments explained below.

Patient-reported outcome measures (PROMs)

Touchscreen questionnaires were used for PROMs at the inclusion visit. The Bath Ankylosing Spondylitis Disease Activity Index (BASDAI) is the patient's evaluation of disease activity. The BASDAI consists of a 6-item scale to measure severity of fatigue, spinal and peripheral joint pain, localised tenderness, morning stiffness (duration and severity), using the VAS scale (0-100 mm). To give each symptom equal weighting, the average of the two scores relating to morning stiffness is taken. The resulting 0 to 500 score is divided by 5 to give a final 0-100 BASDAI score. Scores of 40 or greater indicate suboptimal control of disease (12). Physical function was addressed using the Bath Ankylosing Spondylitis Functional Index (BASFI). The BASFI is a 10-item scale on which respondents rate the degree of difficulty they have in performing certain tasks, using visual analogue scales (VAS) from 0 (easy) to 100 (impossible). The mean of the 10 responses is the BASFI score (13). Pain was assessed by a Visual Analogue Scale (VAS) pain last week/spine/at night/due to AxSpA on a 0-100 mm scale. The intensity of fatigue was assessed by a VAS scale (0-100 mm). Health-related quality of life (HRQoL) was addressed using the Medical Outcomes Study SF-36; SF-36 is a generic health status questionnaire that was developed as a tool to compare various aspects of health status across a general and broad patient population (20-22). The SF-36 examines eight general health domains: physical functioning, role limitations due to physical health problems, bodily pain, general health perceptions, vitality, social functioning, role limitations due to emotional problems and mental health. Furthermore, a physical and mental component summary score will be calculated. We used the Danish version of SF-36 (23) which uses a 4-week recall period. The PGA was assessed by a single question with a range from 0 to 100 mm (VAS).

Statistical Analysis Plan (SAP)

Clinical examination

Spinal mobility was addressed by the BASMI. The BASMI is a composite index of spinal mobility, and is used internationally in research and clinical practice and is recommended by the ASAS (24). RAA performed a 44 swollen/tender joint count, a SPARCC enthesitis score (25) and a tender point count (26).

Para-clinical assessment

Blood samples as specified in the protocol was collected by a trained laboratory technician and treated according to set procedures. Results on inflammation (C-reactive protein, Plasma Calprotectin), Human Leukocyte Antigen B27 (HLA-B27) and faecal calprotectin will be presented in this study.

Analysis methods

This study was designed as a descriptive cross-sectional study. The characteristics of the participants will be described for each gender and axSpA classification using descriptive statistics. Means and SDs or medians and IQRs and Binary outcomes will be presented as numbers and corresponding percentages. The primary variable of interest is BASDAI coded as a continuous variable. The primary analyses will be based on General Linear Models (GLM), investigating if there is an association between BASDAI and each of the covariates of interest (gender and axSpA classification). Furthermore, a test for interaction between gender and axSpA classification will be performed. The model will include gender, axSpA classification and interaction between them as fixed effects. Categorical outcome measures will be analysed with the use of logistic regression with the same fixed effects. A sensitivity analysis will be performed to assess the robustness of the primary analyses, by imputation missing data with the grand mean. Secondary analyses will include the same model using the secondary outcome measures (see **Table 1**).

The relationship between the continuous measure of tender point count and the patient-reported disease activity score (BASDAI) will be analysed by scatter plots with Spearman rank correlation coefficients. We will stratify on gender (**Figure 2A)**, and axSpA classification (**Figure 2B**) (Appendix A). All models will be tested for their assumptions.

Missing data

The number of participants with missing observations will be reported for each outcome variable. We will use a simple manual imputation by replacing missing data with the grand mean of the variables.

Spydergrams

The medical outcomes survey short form 36 (SF- 36) is a validated patient-reported outcome measure of health related quality of life. It includes 35 questions combined into eight domains, and a transition question; domains are summarised into physical component (PCS) and mental component (MCS) summary scores from 0 – 50 where 50 is considered normative (20-22).

The presentation and interpretation of health-related quality of life from SF-36 is complex, and the impact of patterns of disease can be difficult to evaluate (27). We will use 'Spydergrams' which provide a visual method to examine the eight domains of health-related quality of life scored from 0 – 100 simultaneously in a single figure (27); using spydergrams will enable us to compare the impact on health related quality of life of axSpA patients, specifically in terms of how axSpA classification differentially affects aspects of physical, mental and social wellbeing (**Figure 3**).

Statistical software

The analyses will be conducted using STATA (version 15). The program code for the primary analysis in the statistical program:

```
.glm BASDAI i.Gender i.axSpA-classification, family (gamma) link (identity)

.glm BASDAI i.Gender##i.axSpA-classification, family (gamma) link (identity)
```

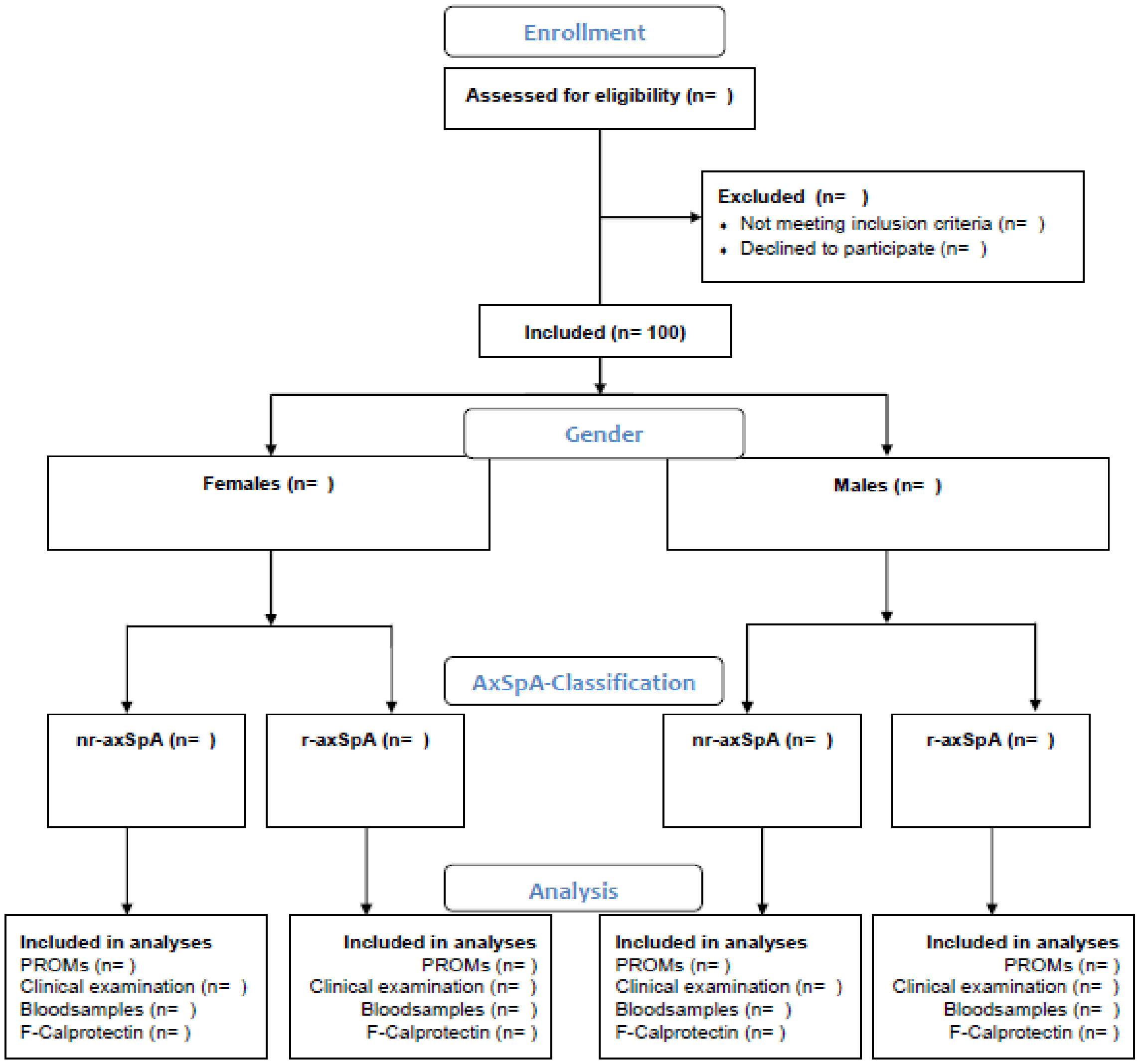

Figure 1. Numbers of axSpA patients who were screened, included in the study, and included in the analyses. Flow chart template modified from http://consort-statement.org.

Statistical Analysis Plan (SAP)

Table 1. Clinical characteristics and patient reported outcomes

	**r-axSpA n =**		**nr-AxSpA n =**			
**Demographics**	Males, n =	Females, n =	Males, n =	Females, n =	Effect of gender	Effecf of axSpA classification
Age, years						
BMI, kg/m^2						
Smoking (current), n (%)						
AxSpA symptom month						
Peripheral involvement, n (%)						
**Co-morbidities**						
Charlson Co-morbidity Index (CCI)						
CCI = 0, n (%)						
CCI = 1, n (%)						
CCI ≥ 2, n (%)						
**Medication**						
NSAIDs daily use, n (%)						
MTX use, n (%)						
MTX dose (mg/week)						
Sulfasalazine use, n (%)						
Sulfasalazine dose (mg/daily)						
No. of previous bDMARD used (if any)						
Current bDMARD, n (%)						
Glucocorticoid use, n (%)						
Glucocorticoid dose (mg/daily)						
**Patient reported outcome measures (PROMs)**						
BASDAI (0-100)						
BASFI (0-100)						
VAS pain due to AxSpA (0-100 mm)						
VAS fatigue (0-100 mm)						
VAS global (0-100 mm)						
SF-36: MCS (0-100)						
SF-36: PCS (0-100)						
**Clinical examination**						
Swollen joint count (0-44)						
Tender joint count (0-44)						
BASMI (0-100)						
Tender point count (0-18)						
SPARCC enthesitis score (0-16)						
ASDAS-CRP						
VAS physician (0-100)						
**Extra-articular manifestations***						
Uveitis, n (%)						
Inflammatory bowel disease, n (%)						
Psoriasis, n (%)						
Dactylitis, n (%)						
Heel enthesitis, n (%)						
Nephrolithiasis, n (%)						
**Paraclinic assessment**						
HLA-B27 positive, n (%)						
CRP (mg/L)						
P-Calprotectin (μ g/ml)						
F-calprotectin (mg/L)						
**Urine samples****						
U-Sodium, U-Oxalate, U-Chloride, U-albumin, U-Calcium, U-Potassium						

BMI, Body Mass Index; NSAIDs, Nonsteroidal anti-inflammatory drugs; MTX, Methotrexate; bDMARD, biologic disease modifying drugs; BASDAI, Bath Ankylosing Spondylitis Disease Activity Index; BASFI, Bath Ankylosing Spondylitis Functional Index; VAS, Visual Analogue Scale; SF-36: MCS/PCS, Medical Outcomes Study Short Form 36 Mental/Physical Component Summary; BASMI, Bath Ankylosing Spondylitis Metrology Index; SPARCC, Spondyloarthritis Research Consortium of Canada; ASDAS-CRP, Ankylosing Spondylitis Disease Activity Score - C-eactive Protein; *either patient history or current diagnosis; P/F-Calprotectin, Plasma/Faecal Calprotectin; *Urine samples will be collected in this study, but will be presented in a seperate future paper. The primary analyses will be based on General Linear Models (GLM), investigating if there is an association between BASDAI and each of the covariates of interest (gender and AxSpA classification). Furthermore, a test for interaction between gender and axSpA classification will be performed. The model will include gender, axSpA classification and interaction between them as fixed effects. Categorical outcome measures will be analysed with the use of logistic regression with the same fixed effects. Secondary analyses will include the same model using the secondary outcome measures

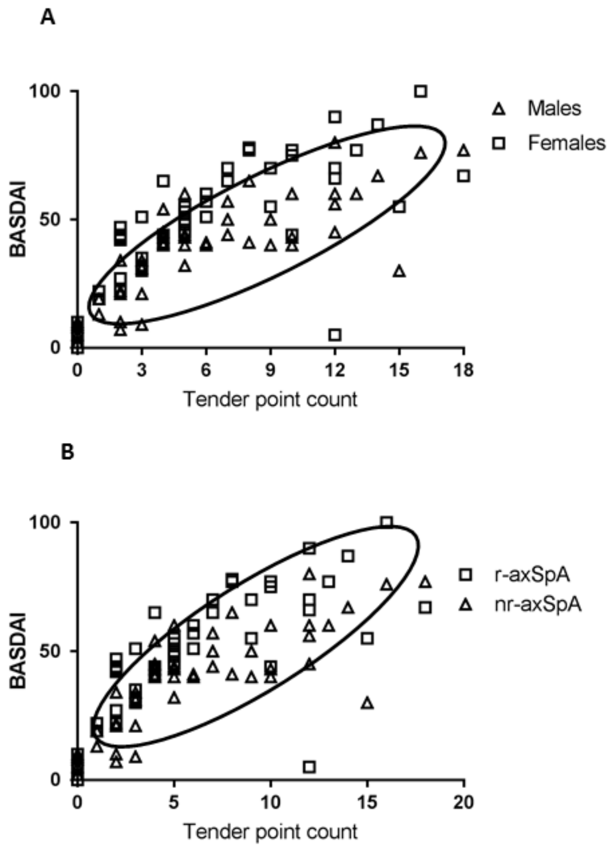

Figure 2. (Data are simulated for visual illustration) Correlation between tender point count and BASDAI. (A) Stratified on gender (B) Stratified on axSpA classification.

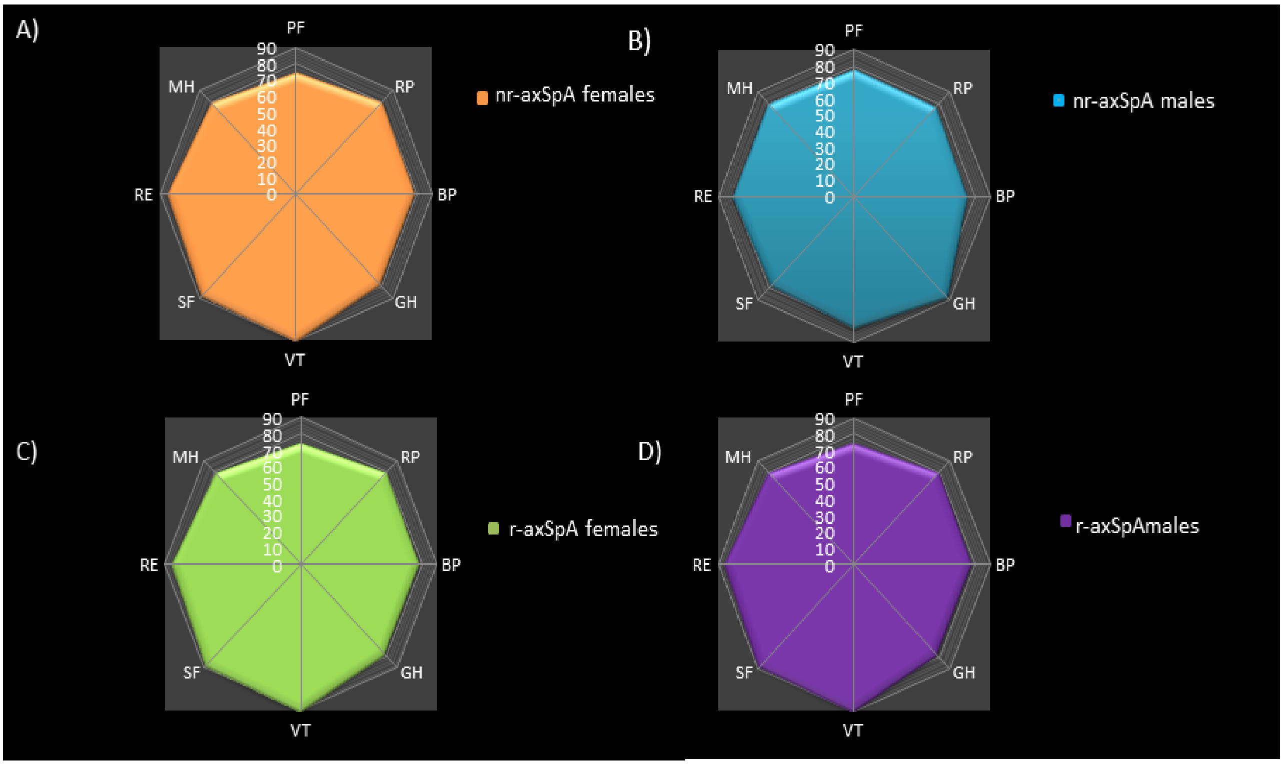

Figure 3 (Data are simulated for visual illustration). Mean SF-36 scores are shown for axSpA patients. (A) nr-axSpA females (B) nr-axSpA males (C) r-axSpA females (D) r-axSpA males. PF, physical function; RP, role physical; BP, bodily pain; GH, general health; VT ,vitality; SF, social functioning; RE, role emotional; MH, mental health.

1. Vandenbroucke JP, von Elm E, Altman DG, Gotzsche PC, Mulrow CD, Pocock SJ, et al. Strengthening the Reporting of Observational Studies in Epidemiology (STROBE): Explanation and elaboration. International journal of surgery (London, England). 2014;12(12):1500-24.
2. Braun J, Kiltz U, Baraliakos X, van der Heijde D. Optimisation of rheumatology assessments - the actual situation in axial spondyloarthritis including ankylosing spondylitis. Clinical and experimental rheumatology. 2014;32(5 Suppl 85):S-96-104.
3. Bremander A, Petersson IF, Bergman S, Englund M. Population-based estimates of common comorbidities and cardiovascular disease in ankylosing spondylitis. Arthritis Care Res (Hoboken). 2011;63(4):550-6.
4. Essers I, Stolwijk C, Boonen A, De Bruin ML, Bazelier MT, de Vries F, et al. Ankylosing spondylitis and risk of ischaemic heart disease: a population-based cohort study. Annals of the rheumatic diseases. 2016;75(1):203-9.
5. Chen HH, Yeh SY, Chen HY, Lin CL, Sung FC, Kao CH. Ankylosing spondylitis and other inflammatory spondyloarthritis increase the risk of developing type 2 diabetes in an Asian population. Rheumatol Int. 2014;34(2):265-70.
6. Zochling J, Smith EU. Seronegative spondyloarthritis. Best practice & research Clinical rheumatology. 2010;24(6):747-56.
7. Rudwaleit M. New approaches to diagnosis and classification of axial and peripheral spondyloarthritis. Current opinion in rheumatology. 2010;22(4):375-80.
8. Roussou E, Sultana S. Spondyloarthritis in women: differences in disease onset, clinical presentation, and Bath Ankylosing Spondylitis Disease Activity and Functional indices (BASDAI and BASFI) between men and women with spondyloarthritides. Clinical rheumatology. 2011;30(1):121-7.
9. Rusman T, van Vollenhoven RF, van der Horst-Bruinsma IE. Gender Differences in Axial Spondyloarthritis: Women Are Not So Lucky. Current rheumatology reports. 2018;20(6):35.
10. Gran JT, Ostensen M, Husby G. A clinical comparison between males and females with ankylosing spondylitis. The Journal of rheumatology. 1985;12(1):126-9.
11. de Carvalho HM, Bortoluzzo AB, Goncalves CR, da Silva JA, Ximenes AC, Bertolo MB, et al. Gender characterization in a large series of Brazilian patients with spondyloarthritis. Clinical rheumatology. 2012;31(4):687-95.
12. Garrett S, Jenkinson T, Kennedy LG, Whitelock H, Gaisford P, Calin A. A new approach to defining disease status in ankylosing spondylitis: the Bath Ankylosing Spondylitis Disease Activity Index. The Journal of rheumatology. 1994;21(12):2286-91.
13. Calin A, Garrett S, Whitelock H, Kennedy LG, O'Hea J, Mallorie P, et al. A new approach to defining functional ability in ankylosing spondylitis: the development of the Bath Ankylosing Spondylitis Functional Index. The Journal of rheumatology. 1994;21(12):2281-5.
14. Jensen OK, Callesen J, Nielsen MG, Ellingsen T. Reproducibility of tender point examination in chronic low back pain patients as measured by intrarater and inter-rater reliability and agreement: a validation study. BMJ open. 2013;3(2).
15. Christensen R, Bliddal H, Henriksen M. Enhancing the reporting and transparency of rheumatology research: a guide to reporting guidelines. Arthritis research & therapy. 2013;15(1):109.
16. Braun J, van den Berg R, Baraliakos X, Boehm H, Burgos-Vargas R, Collantes-Estevez E, et al. 2010 update of the ASAS/EULAR recommendations for the management of ankylosing spondylitis. Annals of the rheumatic diseases. 2011;70(6):896-904.
17. van der Linden S, Valkenburg HA, Cats A. Evaluation of diagnostic criteria for ankylosing spondylitis. A proposal for modification of the New York criteria. Arthritis and rheumatism. 1984;27(4):361-8.
18. Andreasen R, Christensen R, Kristensen L, Ellingsen T, Horn H, Hansen I. FRI0460 Extra-articular manifestations in patients with axial spondyloarthritis: a cross sectional study from southern denmark. Annals of the rheumatic diseases. 2017;76(Suppl 2):660-1.
19. Charlson ME, Pompei P, Ales KL, MacKenzie CR. A new method of classifying prognostic comorbidity in longitudinal studies: Development and validation. J Chronic Dis. 1987;40(5):373-83.

20. McHorney CA, Ware JE, Jr., Lu JF, Sherbourne CD. The MOS 36-item Short-Form Health Survey (SF-36): III. Tests of data quality, scaling assumptions, and reliability across diverse patient groups. Medical care. 1994;32(1):40-66.
21. McHorney CA, Ware JE, Jr., Raczek AE. The MOS 36-Item Short-Form Health Survey (SF-36): II. Psychometric and clinical tests of validity in measuring physical and mental health constructs. Medical care. 1993;31(3):247-63.
22. Ware JE, Jr., Sherbourne CD. The MOS 36-item short-form health survey (SF-36). I. Conceptual framework and item selection. Medical care. 1992;30(6):473-83.
23. Bjorner JB, Thunedborg K, Kristensen TS, Modvig J, Bech P. The Danish SF-36 Health Survey: translation and preliminary validity studies. Journal of clinical epidemiology. 1998;51(11):991-9.
24. Jenkinson TR, Mallorie PA, Whitelock HC, Kennedy LG, Garrett SL, Calin A. Defining spinal mobility in ankylosing spondylitis (AS). The Bath AS Metrology Index. The Journal of rheumatology. 1994;21(9):1694-8.
25. Maksymowych WP, Mallon C, Morrow S, Shojania K, Olszynski WP, Wong RL, et al. Development and validation of the Spondyloarthritis Research Consortium of Canada (SPARCC) Enthesitis Index. Annals of the rheumatic diseases. 2009;68(6):948-53.
26. Wolfe F, Smythe HA, Yunus MB, Bennett RM, Bombardier C, Goldenberg DL, et al. The American College of Rheumatology 1990 Criteria for the Classification of Fibromyalgia. Report of the Multicenter Criteria Committee. Arthritis and rheumatism. 1990;33(2):160-72.
27. Strand V, Crawford B, Singh J, Choy E, Smolen JS, Khanna D. Use of "spydergrams" to present and interpret SF-36 health-related quality of life data across rheumatic diseases. Annals of the rheumatic diseases. 2009;68(12):1800-4.

Appendix A

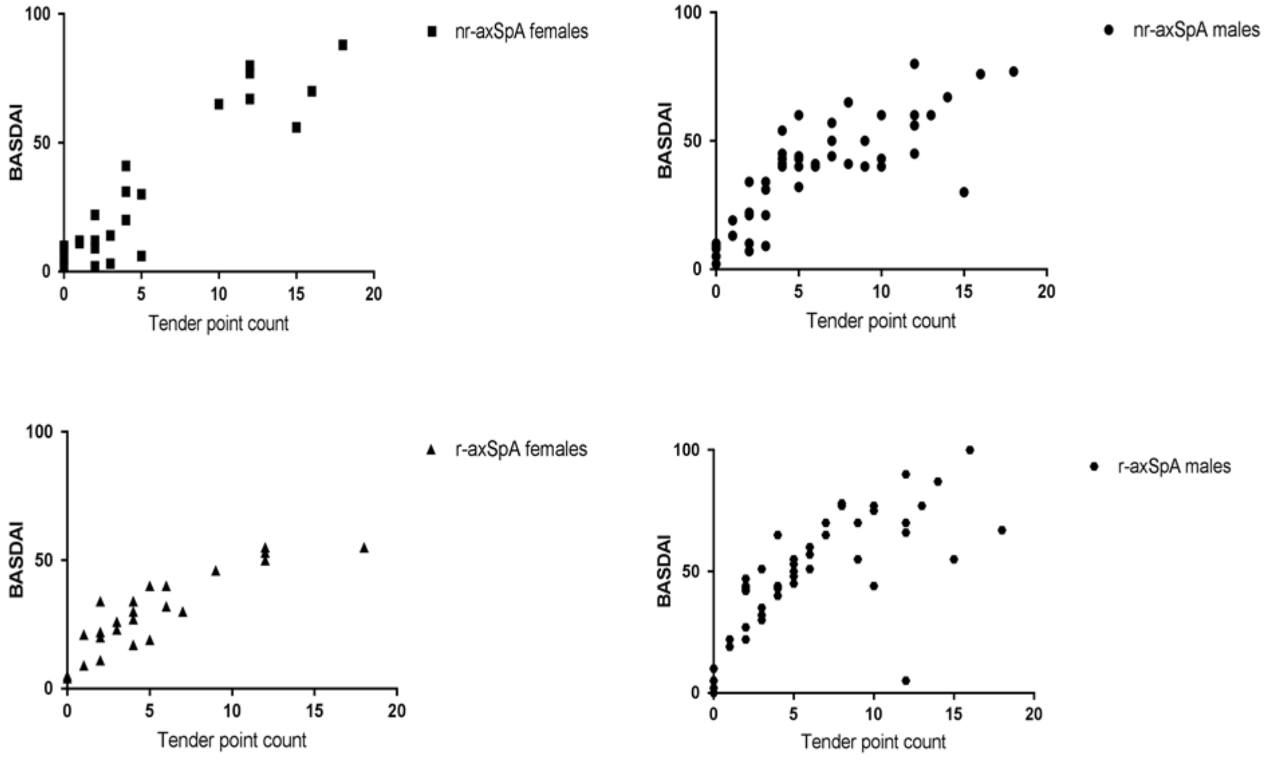

Appendix A. (Data are simulated for visual illustration) Correlation between tender point count and BASDAI stratified on gender and axSpA classification.
